# Primary cilia-mediated regulation of microglial secretion in Alzheimer’s disease

**DOI:** 10.3389/fmolb.2023.1250335

**Published:** 2023-10-23

**Authors:** Seungeun Yeo, Jaemyung Jang, Hyun Jin Jung, Hyeyoung Lee, Youngshik Choe

**Affiliations:** ^1^ Korea Brain Research Institute, Daegu, Republic of Korea; ^2^ Division of Applied Bioengineering, Dong-eui University, Busan, Republic of Korea

**Keywords:** microglia, primary cilia, extracellular vesicles, amyloid-beta, Alzheimer’s disease

## Abstract

Alzheimer’s disease (AD) is a brain disorder manifested by a gradual decline in cognitive function due to the accumulation of extracellular amyloid plaques, disruptions in neuronal substance transport, and the degeneration of neurons. In affected neurons, incomplete clearance of toxic proteins by neighboring microglia leads to irreversible brain inflammation, for which cellular signaling is poorly understood. Through single-cell transcriptomic analysis, we discovered distinct regional differences in the ability of microglia to clear damaged neurites. Specifically, microglia in the septal region of wild type mice exhibited a transcriptomic signature resembling disease-associated microglia (DAM). These lateral septum (LS)-enriched microglia were associated with dense axonal bundles originating from the hippocampus. Further transcriptomic and proteomic approaches revealed that primary cilia, small hair-like structures found on cells, played a role in the regulation of microglial secretory function. Notably, primary cilia were transiently observed in microglia, and their presence was significantly reduced in microglia from AD mice. We observed significant changes in the secretion and proteomic profiles of the secretome after inhibiting the primary cilia gene intraflagellar transport particle 88 (Ift88) in microglia. Intriguingly, inhibiting primary cilia in the septal microglia of AD mice resulted in the expansion of extracellular amyloid plaques and damage to adjacent neurites. These results indicate that DAM-like microglia are present in the LS, a critical target region for hippocampal nerve bundles, and that the primary ciliary signaling system regulates microglial secretion, affecting extracellular proteostasis. Age-related primary ciliopathy probably contributes to the selective sensitivity of microglia, thereby exacerbating AD. Targeting the primary ciliary signaling system could therefore be a viable strategy for modulating neuroimmune responses in AD treatments.

## Graphical Abstract

**Figure:**
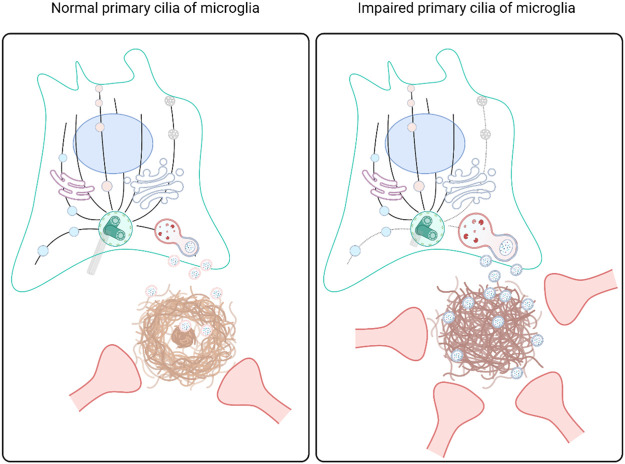
Microglial phagocytosis and exocytosis of Aβ are mediated by primary cilia, which influence extracellular proteostasis, Aβ plaque formation, and neurite dystrophy. When microglia in wild-type mice develop into the disease-associated state, they require genes involved in primary ciliogenesis and extracellular vesicle secretion. Inhibition of Ift88, a key cilia gene, in microglia stimulates phagocytosis and the secretion of extracellular vesicles with an altered proteomic composition, leading to the failure of extracellular proteostasis and neurite degeneration.

## Introduction

Microglia are the phagocytic cells involved in brain development and homeostasis with synapse pruning, clearance of cellular and synaptic debris, and dead cells (Schafer and Stevens, 2015). They mediate age-related primary pathogen defense and responses to injuries and coordinate the cellular crosstalk by secreting cytokines and chemokines (Damani et al., 2011). Microglial secretory vesicles during cellular communication also have important roles in the pathogenesis of neurodegenerative diseases such as Alzheimer’s disease (AD) (Martin et al., 2017; Muraoka et al., 2021). Amyloid-beta (Aβ) plaques recruit activated forms of microglia, also known as disease-associated microglia (DAM) or neurodegenerative microglia (MGnD) (Keren-Shaul et al., 2017; Krasemann et al., 2017). The DAM exhibit promoted phagocytosis of Aβ and dystrophic neurites and condensation of the plaques. Another important genetic feature of DAM is the high expression of genes related to extracellular vesicle (EV) secretion. EVs are membranous vesicles containing a variety of cargo molecules, such as Aβ and phosphorylated tau (p-tau), in AD brains (Muraoka et al., 2020; Cohn et al., 2021). A disease-associated subset of microglia adjacent to Aβ plaques encapsulates and spreads p-tau in TSG101-positive EVs (Clayton et al., 2021). EVs are released from the intracellular endosome recycling pathways, including the multivesicular body and lysosomes. Exocytosis of lysosomal contents is regulated by a complex set of signaling pathways that involve the activation of small GTPases, and the formation of multi-vesicular bodies (MVBs) and subsequent secretions as exosomes (Andrews, 2000; Medina et al., 2011; Krause et al., 2022). The activation of Transcription Factor EB (TFEB), a master regulator of lysosomal biogenesis, leads to the upregulation of the expression of lysosomal proteins and enzymes (Settembre et al., 2012), which can be sorted into MVBs and exosomes. These vesicles are then transported to the cell surface and released by exocytosis. Undegraded Aβ in phagocytic cells is released after lysosomal concentration (Hu et al., 2009). The exocytosis of lysosomal contents serves to remove the phagocytic load from the cell and prevent damage to the phagocytic cell, while simultaneously providing seed for extracellular Aβ plaques and neurite dystrophy. Phagocytosis of extracellular debris or pathogens forms phagolysosomes that consume lysosomal hydrolases, activating a series of enzymatic reactions. Successful lysosomal degradation and clearance occur via the fusion of LC3-marked autophagosomes with lysosomes (Gotzl et al., 2018; Ye et al., 2020). After degradation in the lysosome, substrates are transferred into the cytoplasm and recycled to produce cellular components that serve as substrates in biosynthetic pathways. The promoted phagocytic activity combined with weakening degradative lysosomes impacts cellular viability through undegraded accumulation. Thus, vesicle secretion pathways are well-coupled into the lysosomal function (Hu et al., 2015; Higuchi-Sanabria et al., 2020). Secretion of vesicles containing lysosome contents benefits from overloading lysosome function, bypassing lysosomal storage failure, the formation of toxic accumulates, aberrant activation of signaling pathways, and blockage of the endosome-lysosome pathway (Parenti et al., 2021).

Primary cilia are cellular extrusions composed of microtubule-associated signaling molecules involved in Shh, Notch, PDGF, and Wnt signaling pathways in most cells (Wheway et al., 2018). Pivotal developmental processes are mediated via restricted expression and specific transport of signaling molecules in primary cilia, including cell proliferation, migration, and differentiation (Guemez-Gamboa et al., 2014; Hasenpusch-Theil and Theil, 2021). Mis-localization of signaling molecules in primary cilia is thus tightly linked with neurodevelopmental diseases such as Joubert syndrome, Meckel syndrome, and Bardet-Biedl syndrome (Delous et al., 2007; Berbari et al., 2008). The correct localization of ciliary proteins crucially relies on the intraflagellar transport (IFT) system. The cytoplasmic IFT proteins also mediate extraciliary functions, including autophagy (Pampliega and Cuervo, 2016) and intracellular trafficking of vesicles (Hsiao et al., 2012). The IFT system is associated with endosomal compartments and mediates vesicular trafficking-related functions in leukocytes that do not have a primary cilium (Finetti et al., 2014; Onnis et al., 2016; Vivar et al., 2016). In non-ciliated cells, the IFT system participates in lysosome biogenesis and autophagosome activation by transporting acid hydrolases to lysosomes, recruiting the core autophagy protein ATG16L1 to the early autophagosome, and upregulating TFEB-dependent lysosomal gene network expression (Finetti et al., 2020; Finetti et al., 2021). Primary cilia are positioned near Golgi stacks, which supply Golgi-derived vesicles to replenish the ciliary constituents, including membrane proteins and lipids (Kim et al., 2014; Conduit and Vanhaesebroeck, 2020). Golgi-derived vesicles are not only sorted to the primary cilia but also fused to the MVB before secretion through the plasma membrane or degradation by fusion with the lysosome (Teng and Fussenegger, 2020). Primary cilia also secrete cilia-derived vesicles named ectosomes through the tip of the structure (Mohieldin et al., 2021). However, MVB is the main hub of vesicles for secretion via EVs and degradation by fusion with lysosomes. The primary cilia rooted in the basal body (Stinchcombe et al., 2015), a microtubule organizing and synthesizing center, should be directly or indirectly involved in the control of vesicular trafficking, including the MVB. Vesicle trafficking to the primary cilia depends on the microtubule network (Pedersen et al., 2016), however, it has not been studied whether the ciliary trafficking system is linked to the biosynthesis, transport, and secretion of MVB. Inhibition of Ift88, a protein that plays an essential role in cilium biogenesis, shortens cilia length and affects exocytosis, including vesicle number and proteomic contents of the vesicles (Zuo et al., 2019; Mohieldin et al., 2021). The coupling of cytosolic exocytosis and signaling through the primary cilia should be critical, especially for microglia, in which secretion occurs in a toxic milieu that could affect the primary cilia signaling. The coordinated alignment of cells within a plane facing the pathologically misfolded protein aggregate may play a crucial role in achieving specific functions, such as the removal or compaction of toxic substrates. The primary cilia are well-position to control the coordinated migration of phagocytic cells toward the Aβ plaques, which has not been addressed. The secretion and endocytosis from the microglial processes could be under the control of the primary cilia in the perinuclear compartment, closely located to the enlarged lysosome. The primary cilia facing the extracellular milieu may be involved in maintaining extracellular proteostasis by regulating phagocytosis, lysosomal clearance, and protein loading and secretion of EVs in microglia. In this study, we demonstrated that Ift88 in the microglia is an essential component of the vesicle trafficking between the lysosome and EV secretion. The impaired Ift88 function of microglia caused the secretion of EVs containing altered proteomic contents, which exacerbated extracellular proteostasis and promoted the growth of Aβ plaques and related neurite dystrophy in AD model mice.

## Materials and methods

### Animals

All animal experiments were conducted in accordance with the ethical guidelines of the Korea Brain Research Institute and with the approval of the Korea Brain Research Institute Ethical Committee for Animal Experimentation (approved protocol numbers IACUC-22-00014, M2-IACUC-21-00014). Mice were housed in standard ventilated cages with *ad libitum* access to food and water on a standard 12-h light/12-h dark cycle. 5xFAD (also known as Tg6799, B6SJL-Tg(APPSwFlLon, PSEN1*M146L*L286V)6799Vas/Mmjax), Ift88-floxed (B6.129P2-Ift88tm1Bky/J), and Cx3cr1-cre (B6J.B6N(Cg)-Cx3cr1tm1.1(cre)Jung/J) mice were purchased from the Jackson Laboratory and C57BL/6 mice via Orient Bio. (South Korea). Adult 5xFAD and their respective Ift88-flox/flox littermates were utilized in the experiments. To examine the effect of the loss of function of the Ift88 allele in a 5xFAD background, 5xFAD; Ift88-flox/flox and 5xFAD; Ift88-flox/wt mice were used.

### AAV vector construction

A recombinant AAV vector was created by combining two different plasmids: pAAV-CD68-hM4D(Gi)-mCherry, a gift from Dr. Bryan Roth (Addgene plasmid #75033), and pAAV-GFP/Cre, a gift from Dr. Fred Gage (Addgene plasmid, #49056). The EGFP-Cre fragment was amplified using a forward primer (CCG​CGG​GTC​GAC​GCC​ACC​ATG​GTG​AGC​AAG​GGC​GA) and a reverse primer (CCG​CCC​GAA​TTC​CTA​ATC​GCC​ATC​TTC​CAG​CA) using pAAV-GFP/Cre as a template. The pAAV-CD68-Cre plasmid was generated by inserting a *SalI-EcoRI* fragment containing the EGFP-Cre into the pAAV-CD68-hM4D(Gi)-mCherry after removal of hM4D(Gi)-mCherry using *SalI* and *EcoRI* sites. The pAAV-pCD68-Cre was propagated in Stbl3 *E. coli* cells (Invitrogen™ One Shot™ Stbl3™ Chemically Competent *E. coli*, C737303, ThermoFisher Scientific) and sequenced (Bionics) to check for correct expression.

### AAV purification

Generation and purification of AAV-PHPeB-pCD68-Cre and AAV1 (AAV-hSyn1-EGFP-P2A-EGFPf-WPRE-HGHpA, a plasmid generously provided by Dr. Guoping Feng from Addgene, plasmid #74513) were conducted following established protocols provided by Addgene. In brief, AAV vectors, rep/cap packaging plasmids, and adenoviral helper plasmids were added to AAV-293 cells after mixing with polyethyleneimine (PEI, Polysciences, Inc.). After 72 h of transfection, the supernatant was collected and precipitated overnight at 4°C by mixing it with 40% w/v PEG-8000 and 0.4M NaCl. The resulting mixture was centrifuged at 2,500 *g* for 30 min at 4°C, and the pellet was resuspended in PBS containing 0.001% Pluronic F68, and 200 mM NaCl (virus buffer). Simultaneously, the cell pellet was resuspended in virus buffer and sonicated with four 1-s pulses, followed by at least 15 min of incubation on ice. The cell debris was pelleted at 3,220 g for 15 min, and the clear lysate was transferred to the tube that contained the resuspended virus. The total crude virus was subjected to iodixanol gradient centrifugation. Recovered AAVs were concentrated, and the buffer was exchanged with virus buffer using Amicon 100K columns (EMD Millipore). To determine virus titers, RT-PCRs were performed.

### Stereotaxic AAV injection

Mice aged 6 months were fasted for 12 h prior to surgery. For anesthesia, 150 mg/kg of 2,2,2-tribromoethanol (Sigma-Aldrich) was administered, and 1.5 percent isoflurane in combination with oxygen was used to maintain deep anesthesia. The animals were placed in a stereotaxic frame (Kopf Instruments), and the surgical site on the head was cleaned with isopropyl alcohol. Stereotaxic coordinates were determined based on the location of the bregma. Following a minor incision to expose the skull, small holes with a diameter of less than 1 mm were drilled bilaterally for virus injection. Each animal received approximately 1.5 × 10^9 viral genomes of AAV1 (AAV-hSyn1-EGFP-P2A-EGFPf-WPRE-HGHpA) injected at a rate of 30 nL/min into specific regions: the mPFC (bregma +2.0, ±0.45, dura −1.7), vDG (vDG, lambda. 0.4, 2.9, dura-2.7), and the LS (bregma. 0.4, 0.3, dura-2.6). The injection was performed using a pulled glass pipette (20–30 µm inner diameter) controlled by a Nanoject III (Drummond Scientific) to regulate the injection speed. Following the procedure, the incision was sutured, and the mice were returned to their respective home cages once they had fully recovered from anesthesia. Post-operative care, including buprenorphine injections, was provided as required by the IACUC of Korea Brain Research Institute. One month after AAV injection, brain tissues were collected and perfused with 4% paraformaldehyde for subsequent immunostaining and three-dimensional imaging.

### Tissue clearing and visualization of AAV tracers

The X-Clarity tissue clearing system II (Logo Biosystems, Korea) was employed to achieve brain tissue transparency in mouse brains 14 days after AAV injection. The brains were polymerized for a period of 3 hours and subsequently cleared for 24 hours using the X-Clarity system. Following tissue clearing, brain samples were subjected to antibody staining utilizing the DeepLabel Antibody Staining Kit (Logos Biosystems, Korea). After thorough washing with PBST, the tissues were incubated with anti-GFP antibodies (1:200) in 0.1% tween 20 in PBS at 37°C for a duration of 1-2 days. This was followed by an additional day of washing and subsequent incubation with secondary antibodies conjugated with Alexa Fluor 488 (1:400, Invitrogen, United States). To facilitate imaging, the cleared brain tissues were immersed in an RI matching solution for 24–48 h, consisting of 50% sucrose and 25% urea. For three-dimensional circuit visualization using AAV tracers, the cleared samples were imaged using a light sheet fluorescence microscope (Ultramicroscope II, LaVision BioTec GmbH, Germany) equipped with a fiber laser source (SuperK EXTREME EXW-12, NKT photonics A/S, Denmark) to generate the light sheet. The emitted light was transmitted through a 2x objective lens (MVPLAPO, Olympus, Japan) and detected by a Neo sCMOS camera (ANDOR NEO, Oxford Instruments, United Kingdom). The GFP signals of the AAV tracers were observed using a bandpass filter set with an excitation range of 470/40 nm and an emission range of 520/50 nm. The numerical aperture of the light sheet was calculated to be 0.073 in the system software, and the thickness of the light sheet was approximately 5 μm at the manufacturer’s setting. Since the axial resolution to separate two different structures parallel to the laser beam was determined by the numerical aperture of the light sheet system, the step size in the *Z*-direction between each image was set to 5 µm. The resulting serial TIFF image files were subsequently converted to the Imaris file format, enabling image post-processing and three-dimensional rendering using Imaris software (Bitplane, Cologne).

### Cell culture

BV2 cells (kindly provided by Dr. Hoe, Hyang-Sook) were maintained in DMEM high glucose (Thermo Fisher) supplemented with 10% fetal bovine serum (Thermo Fisher Scientific) and 1% penicillin/streptomycin (Thermo Fisher Scientific). Subculturing of the cells was performed every 3 days, and the cells were incubated at 37°C in a humidified atmosphere with 5% CO_2_.

### Preparation of Aβ (1-42) and pHrodo-Aβ (1-42) for cell treatment

Aβ 1-42 peptide (Bachem AG) was dissolved in sterile distilled water to achieve a concentration of 500 µM and then incubated at 37°C for 5 days (Pike et al., 1993). For the phagocytosis assay, Aβ (1-42) was labeled with pHrodo using the pHrodo iFL red microscale protein labeling kit (Invitrogen) according to the manufacturer’s instructions.

### Transfection and treatment of Aβ (1-42) or LysoTracker

SiRNA transfection was conducted using Lipofectamine RNAiMAX Transfection Reagent (Thermo Fisher Scientific). BV cells were transfected with 80 nM of mouse siIft88 or a negative control siRNA (Bioneer). The RNA sequence of the mouse siIft88 siRNA used was ACU​GGG​AGA​GUU​AUA​CGA​U. Following siRNA transfection, BV2 cells were treated with a final concentration of 1 μM Aβ (1-42), pHrodo-Aβ (1-42), or 50 nM LysoTracker (Invitrogen).BV2 cells were seeded at a confluence of approximately 80%. The following day, cells were transfected with siRNA and treated with Aβ 6 hours after siRNA transfection. BV2 cells transfected with siRNA were exposed to Aβ for 18 h. BV2 cells were harvested or fixed at 24 h after transfection. For the analysis of the intensity of pHrodo-Aβ and lysotracker, 3 z-stack images (1.5 µm thick) were acquired per experiment using a confocal microscope (100x oil objective, Plan Apo VC 100x Oil D IC N2, Nikon A1/Ni-E). Maximal intensity projection pictures from every z-stack were created using NIS-Elements AR analysis software (Nikon). The intensity of pHrodo-Aβ and lysotracker in each BV2 cell was analyzed using NIS-Elements AR analysis software (Nikon). We pooled data from the 3 technical replicates.

### Immunohistochemistry and confocal microscopy

The mice were perfused with 4% paraformaldehyde and subsequently post-fixed for an additional 3 h. Brain tissues were cryoprotected in a 20% sucrose solution and embedded in OCT compounds to obtain thin sections of 12-20 µm thickness. The tissue sections were then rinsed in PBS containing 0.2% Tween20 (Merck Millipore) for 10 min, followed by overnight incubation at 4 °C with primary antibodies (refer to the antibody table) diluted in 5% BSA. After washing with PBST, the sections were incubated with appropriate fluorescent-conjugated secondary antibodies (refer to the antibody table, Thermo Fisher Scientific) for 4 h at room temperature. To stain EV with PSVue-550, the tissue sections were incubated 10 µM of PSVue-550 (Molecular Targeting Technologies) and Amylo-Glo for 1 h and then washed with washing buffer (pH7.4) including 5 mM TES and 145 mM NaCl. Immunofluorescence images were captured using a Nikon Ai Rsi/Ni-E confocal microscope, and the signals were analyzed using NIS-Elements AR analysis software (Nikon).

A confocal microscope (Nikon A1/Ni-E) with laser sources (Coherent; 403 nm (power 100 mW), 457/488/514 nm (power, 40 mW), 561 nm (power 20 mW), and 640 nm (power 40 mW) were used to capture the images. The bandpass filter configured with an excitation range of 520/50 nm and an emission range of 500/50 nm was used to observe Alexa 488 signals. The bandpass filter configured with an excitation range of 595/50 nm and an emission range of 570/50 nm was used to examine Alexa 594 signals. The bandpass filter configured with an excitation range of 700/75 nm and an emission range of 663/75 nm was used to observe Alexa 647 signals. The bandpass filter was configured with an excitation range of 450/50 nm and an emission range of 400/50 nm was used to detect Amylo-Glo signals.

For the analysis of the axon spheroid near the Aβ plaque in the LS, NAc, CP, and Hb (6-month-old, n = 4/each group, female), 5 z-stack images (1.5 µm thick) were acquired per experiment using a confocal microscope (20x dry objective, Plan Apo VC 20x DIC N2 with 3x digital zoom, Nikon A1/Ni-E). Microcopy acquisition settings were kept constant within the same experiment. Maximal intensity projection pictures from every z-stack were created using NIS-Elements AR analysis software (Nikon). Axon spheroids near each Aβ plaque were counted within the area (300 μm × 300 µm).

To analyze the percentage of AC3-positive microglia and measure the length of AC3 in the LS and cortex (5-month-old, n = 4/each group, female), 10 z-stack images (1 µm thick) were acquired per experiment using a confocal microscope (100x oil objective, Plan Apo VC 100x Oil DIC N2, Nikon A1/Ni-E). Maximal intensity projection pictures from every z-stack were created using NIS-Elements AR analysis software (Nikon). We counted the microglia and measured the length of the AC3 in the area (120 μm × 120 µm), where AC3 was present in the main process of the microglia.

To count the number of Aβ plaques (D54D2) and Amylo-Glo plaques and to measure the area of Aβ plaques (D54D2) and Lamp1 in the LS (10-month-old, n = 3/each group, one male and two females), 2D images were acquired per experiment using a confocal microscope (20x dry objective, Plan Apo VC 20x DIC N2 with 3x digital zoom, Nikon A1/Ni-E). Aβ plaques (D54D2) and Amylo-Glo plaques were counted, and the area of Aβ plaques (D54D2) and Lamp1 was measured within the area (500 μm × 500 µm) using NIS-Elements AR analysis software (Nikon). To get representative images of Aβ plaques (D54D2) and Lamp1, 3 z-stack images (2.5 µm thick) were acquired per experiment using a confocal microscope (100x oil objective, Plan Apo VC 100x Oil DIC N2, Nikon A1/Ni-E). Maximal intensity projection pictures from every z-stack were created using NIS-Elements AR analysis software (Nikon).

To count the number of microglia (10-month-old, n = 3/each group, one male and two females), located within <5 µm distance from the Amylo-Glo plaques in the LS, 3 z-stack images (2.5 µm thick) were acquired per experiment using a confocal microscope (100x oil objective, Plan Apo VC 100x Oil DIC N2, Nikon A1/Ni-E). Maximal intensity projection pictures from every z-stack were created using NIS-Elements AR analysis software (Nikon). Microglia, located at < 5 µm from the Amylo-Glo plaques, were counted in each image. Data from three animals in each group was combined.

To analyze the intensity of EV on each dense plaque stained with Amylo-Glo in the LS and cortex (10-month-old, n = 3/each group, one male and two females), 4 z-stack images (2 µm thick) were acquired per experiment using a confocal microscope (100x oil objective, Plan Apo VC 100x Oil DIC N2, Nikon A1/Ni-E). Maximal intensity projection pictures from every z-stack were created using NIS-Elements AR analysis software (Nikon). We measured the fluorescence intensity of CD63, CD81, or PSVue-550 on each dense plaque stained with Amylo-Glo using NIS-Elements AR analysis software (Nikon). Data from three animals in each group was combined. Information on the antibodies used in each experiment is listed in Table 1.

**TABLE 1:** Information of antibodies used in figures.

	1st-antibody (dilution)	Company	Cat. No	Clonality	2nd-antibody (dilution rate)	Company	Cat. No
Figure 1 B	GFP DyLight 488 (1:200)	Novusbio	NBP1-69963	Mouse	N.A.	N.A.	N.A.
Figures 1C–H	Anti-GFP (1:100)	ThermoFisher Scientific™	A-11120	Mouse	Goat anti-Mouse IgG (H + L) Secondary Antibody, Alexa Fluor^®^ 488 conjugate (1:250)	Life technologies	A11029
	Anti-GFP (1:100)	Novusbio	NBP1-69963	Mouse	Goat anti-Mouse IgG (H + L) Secondary Antibody, Alexa Fluor^®^ 488 conjugate (1:250)	Life technologies	A11029
	Anti-β-amyloid (D54D2) (1:100)	Cell Signaling	8243S	Rabbit	Goat anti-Rabbit IgG (H + L) Secondary Antibody, Alexa Fluor 594 (1:250)	Life technologies	A11037
Figure 4A	Anti-AC3 (1:100)	Abcam	ab125093	Rabbit	Donkey anti-Rabbit IgG (H + L) Highly Cross-Adsorbed Secondary Antibody, Alexa Fluor 488 (1:250)	Life technologies	A21206
	Anti-Iba1 (1:100)	Abcam	ab5076	Goat	Donkey anti-Goat IgG (H + L) Secondary Antibody, Alexa Fluor 594 (1:250)	Life technologies	A11058
	Anti-β-amyloid (1-16) (6E10) (1:100)	BioLegend	803001	Mouse	Donkey anti-Mouse IgG (H + L) Secondary Antibody, Alexa Fluor^®^ 647 conjugate (1:250)	Life technologies	A31571
Figure 8B,C,E,G. Supplementary Figure S5A	Anti-Lamp1 (1D4B) (1:100)	Developmental Studies Hybridoma Bank (DSHB)	1D4B	Rat	Donkey anti-Rat IgG (H + L) Secondary Antibody, Alexa Fluor^®^ 594 conjugate (1:250)	Life technologies	A21209
	Anti-β-amyloid (D54D2) (1:100)	Cell Signaling	8243S	Rabbit	Donkey anti-Rabbit IgG (H + L) Secondary Antibody, Alexa Fluor^®^ 647 conjugate (1:250)	Life technologies	A31573
	Anti-CD63 (1:100)	Sicgen	AB0047-200	Goat	Donkey anti-Goat IgG (H + L), Secondary Antibody, Alexa Fluor^®^ 647 conjugate (1:250)	Life technologies	A21447
	Anti-CD81 (1D6) (1:100)	Novusbio	NBP2-67722	Rabbit	Donkey anti-Rabbit IgG (H + L) Secondary Antibody, Alexa Fluor^®^ 647 conjugate (1:250)	Life technologies	A31573
	Anti-Iba1 (1:100)	Abcam	ab5076	Goat	Donkey anti-Goat IgG (H + L) Secondary Antibody, Alexa Fluor 594 (1:250)	Life technologies	A11058
	Amylo-Glo RTD Amyloid Plaque Stain Reagent (1:1000)	Biosensis	TR-300-AG	N.A.			
	PSVue-550	Molecular Targeting Technologies	P-1005	N.A.			
Supplementary Figure S5C	Anti-GFAP antibody [G-A-5] (Cy3 ^®^) (1:200)	Abcam	Ab49874	Mouse	N.A.	N.A.	N.A.
	Anti-Iba1 (1:100)	Abcam	ab5076	Goat	Donkey anti-Goat IgG (H + L) Secondary Antibody, Alexa Fluor 594 (1:250)	Life technologies	A11058
	Anti-β-amyloid (D54D2) (1:100)	Cell Signaling	8243S	Rabbit	Donkey anti-Rabbit IgG (H + L) Secondary Antibody, Alexa Fluor^®^ 647 conjugate (1:250)	Life technologies	A31573
	Amylo-Glo RTD Amyloid Plaque Stain Reagent (1:1000)	Biosensis	TR-300-AG	N.A.			

### EV preparation

The conditioned medium was collected from BV2 cells 24 h after transfection with siIft88 or siCon and subsequent treatment with Aβ. EVs present in the conditioned medium were isolated using the ExoQuick^®^ ULTRA EV Isolation Kit (SBI System Biosciences) following the manufacturer’s instructions. The precipitated EVs were then resuspended in HBSS buffer for NTA. For Co-IP (co-immunoprecipitation), EV pellets were resuspended in lysis buffer containing 1% Nonidet P-40 (Sigma-Aldrich), 50 mM Tris-HCl, 150 mM sodium chloride (pH 7.4), and Halt™ Protease and Phosphatase Inhibitor Single-Use Cocktail (Thermo Fisher Scientific).

### Nanoparticle tracking analysis (NTA)

NTA was conducted using the NanoSight LM10 instrument (Malvern Instruments) following the manufacturer’s user manual (NanoSight LM10 User Manual). The capture and analysis of the particles were performed using the integrated NanoSight Software NTA3.2 (Malvern Instruments). For optimal particle visibility without signal saturation, the camera level was set to 10. Most observed particles were detected using a detection threshold of 15. The NTA measurements were performed at 25°C, and each sample was measured for 60 s, five times.

### Co-immunoprecipitation (Co-IP)

BV2 cells were seeded at a confluence of approximately 80%. The following day, cells were transfected with siRNA and treated with Aβ 6 hours after siRNA transfection. BV2 cells transfected with siRNA were exposed to Aβ for 18 h.BV2 cells and CM were harvested at 24 h after transfection. Cells and EVs were lysed using a lysis buffer composed of 1% Nonidet P-40 (Sigma-Aldrich), 50 mM Tris-HCl, 150 mM sodium chloride (pH 7.4), and Halt™ Protease and Phosphatase Inhibitor Single-Use Cocktail (Thermo Fisher Scientific). The cell lysates were then subjected to immunoprecipitation by incubating them overnight at 4 C with either anti-CD63 (Sicgen) or anti-CD81 (Novus) antibodies. Subsequently, the mixtures were incubated with Dynabeads Protein G (Thermo Fisher Scientific) for 4 h at room temperature. After several washes with PBST containing 0.01% Tween20 in PBS, the immunoprecipitated proteins bound to the beads were eluted using 2x sample buffer (BioRad) for subsequent western blot analysis, or the beads containing the binding proteins were directly analyzed using LCMS.

### Western blot analysis

The BCA protein assay kit (Thermo Fisher Scientific) was used to access protein concentration before using 15 μg of proteins onto 4%–20% Mini-PROTEAN^®^ TGX™ Precast Protein Gels (Bio-Rad Laboratories). Subsequently, the proteins were transferred to Immobilon®-P PVDF membranes (Millipore). The membranes were blocked for 30 min in a PBS solution containing 5% skim milk (BD Biosciences) and 0.1% Tween 20 (Merck Millipore). Primary antibodies, including anti-CD63 (Sicgen, AB0047-200, 1:1,000), anti-CD81 (Novus Biologicals, NB100-65805, 1:1,000), anti-GAPDH (Origene, TA802519, 1:1,000), anti-Ift88 (Proteintech, 60227-1-Ig, 1:1,000), anti-β-Amyloid (D54D2) (Cell Signaling, 8243S, 1:1,000), anti-TMEM119 (Proteintech, 66948-1, 1:1,000) anti-P2RY12 (BioLegend, 848002, 1:1,000), anti-C1Q (Abcam, ab182451, 1:1,000), anti-SPARC (Cell Signaling, 8725, 1:1,000), anti-αTub (Abcam, ab21058, 1:1,000), anti-Trem2 (Novusbio, nbp1-44067, 1:1,000), anti-CD9 (Bio-Rad, MCA2749, 1:1,000), anti-SPP1(R&D System, AF808, 1:1,000), anti-Dap12 (LSbio, ls-ls-c749774/193791, 1:1,000), anti-Apoe (Abcam, ab 1906, 1:1,000), and anti-β-actin (Origene, TA811000S, 1:1,000), were then incubated with the membranes overnight at 4°C. Following washing steps, HRP-conjugated secondary antibodies were incubated with the membranes for 30 min at room temperature. The HRP signals were visualized using an Enhanced Chemiluminescence Reagent Kit (Thermo Fisher Scientific, United States). Information about the mice used in each experiment is listed in Table 2.

**TABLE 2:** Information of mice used in figures.

	Control	Experiment
Figure 1B	6 months/n = 4/Female	-
Figures 1C–E	6 months/n = 4/Female	6 months/n = 4/Female
Figure 2A	3 months/n = 17,796/Male	3 months/n = 39,577/Male
	6 months/n = 2,642/Female (hippocampus, habenula)	6 months/n = 2,156/Female (hippocampus, habenula)
	6 months/n = 9,934/Male (septum)	6 months/n = 11,018/Male (septum)
	9 months/n = 27,819/Male and female	
	12 months/n = 33,243/Male	
Figure 4B	5m/n = 4/Female	5m/n = 4/Female
Figures 5B,D,E,F	BV2 culture, n = 3	BV2 culture, n = 3
Figure 5H, I	BV2 culture, n = 4	BV2 culture, n = 4
Figure 6	Lysate, EV n = 4	Lysate, EV n = 4
Figure 7	CD63IP_Lysate, EV, n = 4	CD63IP_Lysate, EV, n = 4
	CD81IP_Lysate, EV, n = 4	CD81IP_Lysate, EV, n = 4
Figure 8	10 months/n = 3/1 Male, 2 Female	10 months/n = 3/1 Male, 2 Female

### LCMS analysis

Protein samples and beads containing immunoprecipitated proteins were subjected to reduction by adding 2 μL of 500 mM DL-dithiothreitol and incubated for 30 min at 55°C. Alkylation was then accomplished by adding 4 μL of 500 mM iodoacetamide and incubating at room temperature in the dark for 20 min. Trypsin digestion was carried out overnight at 37°C using an enzyme/substrate ratio of 1:50. For label-free proteomics, the samples were cleaned using a PierceTM C18 spin column (Thermo Fisher Scientific), followed by vacuum drying and storage at −20°C until further use. Prior to analysis, the dried peptides were resuspended in 20 μL of 0.1% formic acid. The dried peptides were resuspended in 20 μL of 0.1% formic acid. Nano-liquid chromatography (UltiMate 3,000, Thermo Fisher Scientific) coupled to a Q-Exactive Plus Orbitrap mass spectrometer (Thermo Fisher Scientific) was utilized for peptide analysis. All analyses utilized a binary solvent system comprising 0.1% formic acid in water and acetonitrile, respectively, as previously described (Jang et al., 2023). Peptide fractions were separated using an Ultimate 3,000 RSLCnano System (Thermo Fisher Scientific) with a PepMap 100 C18 LC column (#164535, Thermo Fisher Scientific) as a loading column, followed by a PepMap RSLC C18 (#ES903, Thermo Fisher Scientific) analytical column using 10%–50% solvent B (0.1% formic acid in ACN) in 80 min, 50%–95% solvent B in 0.1 min, a 20 min hold of 90% solvent B, a return to 10% solvent B in 0.1 min, and finally a 20 min hold of 10% solvent B. All flow rates were 300 nL/min delivered using a Easy-nLC1000 liquid chromatography system (Thermo Scientific). Solvent A consisted of water and 0.1% formic acid. Peptides were ionized with an EASY Spray source (Thermo Scientific) held at 250°C and set to 1.8 kV. For MS1 (Full MS mode), precursor ion selection scanning between 350 and 2,000 m/*z* at a resolution of 70,000, a AGC target of 3e^6^, and a maximum IT of 100 ms in the Orbitrap mass analyzer were utilized. For MS2 (ddMS^2^ mode), precursor ion selection is scanned between 350 and 2,000 m/*z* at a resolution of 17,500. A AGC target of 1e^5^, and a maximum IT of 100 ms in the Orbitrap mass analyzer were utilized.

### Mass spectrometry data processing

Proteome Discoverer™ (PD; version 3.0) software was used for protein identification and quantification. Raw MS data were searched against a protein database obtained from the UniProt Knowledgebase Release 2022_5 (14-Dec-2022). Searches are performed utilizing the preinstalled processing workflow (PWF_Hybrid_Precursor_Quan_and LFQ_SequestHT_Percolator) and consensus workflow (CWF_Comprehensive_Enhanced Annotation _LFQ_and Precursor_Quan) as provided by PD3.0. In the processing workflow steps (PWF_Hybrid_Precursor_Quan_and LFQ_SequestHT_Percolator), peptides are required to have a minimum length of ≥6 and a maximum allowable number of reported peptides equal to 10. In the consensus workflow steps (CWF_Comprehensive_Enhanced Annotation _LFQ_and Precursor_Quan), a minimum of one peptide sequence is used as a protein filter criterion. The Protein FDR Validator node in PD3.0 was used to analyze the protein data (confidence thresholds = 0.01). This node systematically assessed the list of target proteins, calculating the FDR as it progressed from the highest to the lowest-ranked proteins. Subsequently, a protein list consisting of high-confidence candidates (FDR <0.01) was selected for the differential analyses. A protein list with abundance values obtained at FDR <0.01 was used for the downstream differential expression analyses. The abundance values of identified proteins were log-transformed and normalized according to the default algorithms of PD3.0.

Lists of identified peptides with a fold-change value and a *p*-value were exported as a text file. The text file was transferred to the Jupyter environment with the R kernel (version 4.3.0) and analyzed following the analysis workflow in the Differential Enrichment Analysis of Proteomics Data (Zhang et al., 2018). An adjusted *p*-value less than 0.05 and an absolute value of fold change larger than 0.25 were used as cut-off criteria. The abundance values of identified proteins were log-transformed and normalized according to the algorithms of PD3.0. A comparison of differentially expressed proteins between two types of experimental groups is shown in Venn diagrams (Gao et al., 2021). The differential expression protein analysis was visualized in a volcano plot using the ggplot2 package (Wickham, 2016). For further functional pathway enrichment analysis of the differentially expressed proteins, the Enrichr R package with Reactome, KEGG, and GO datasets was employed. The enriched pathways were selected based on a *p*-value <0.01. Cytoscape (version 3.9.1) (Shannon et al., 2003) was used to generate a network of these proteins by mapping protein data onto a PPI network. The network was constructed in Cytoscape’s public database section using the STRING database (Doncheva et al., 2019).

### Sample preparation, library construction, and sequencing

Mice were sacrificed by carbon dioxide gas overdose, and the brains were rapidly dissected out. The hippocampus of 3-, 6-, 9-, and 12-month-old mice, the septum of 6-month-old mice, and the Hb of 3-, 9-, and 12-month-old mice were collected from 5xFAD mice and WT mice. The samples were cut into small pieces, placed in digestion media (Neurobasal media (Gibco) containing 1 mg/mL Collagenase type 1, 2, 4 (Worthington Biochemical Corp., United States) or papain (Worthington Biochemical Corp., United States), and 20 U/mL of DNaseⅠ (Sigma)), and incubated in a shaking incubator at 37 C for 45 min. The digested tissues were mechanically dissociated and filtered through a 40 μm cell strainer. The papain was additionally inactivated by adding 20 μg/mL ovoicoid protease inhibitor (Worthington Biochemical Corp.). The single-cell suspension was centrifuged at 500 *g* for 15 min at 4°C and the resulting pellet was suspended in ice cold neurobasal media containing B27. This neurobasal media wash was repeated twice with two different spins at 100 g and 200 g centrifugation to remove dead cells and cellular debris. The single-cell suspension was finally suspended in an ice-cold Hanks’ balanced salt solution with 0.04% bovine serum albumin (A3294, Sigma-Aldrich, United States) at approximately 2 × 10^5^ cells/mL cell population.

The single cells were profiled using in Drop™ barcoded beads in a home-made microfluidic device connected to the Drop-seq apparatus in the Korea Brain Research Institute. Libraries from isolated single cells were generated according to the in Drop™ Protocol with the following modifications (Zilionis et al., 2017). Amplified cDNAs were not fragmented, and cDNA cleanup and size selection were performed using SPRIselect beads (B23317, Beckman Coulter, United States). Amplified cDNAs and final libraries were assessed on an Agilent BioAnalyzer using the High Sensitivity DNA Kit (Agilent Technologies, United States). The libraries were pooled and sequenced on a NovaSeq 6,000 system or a NextSeq500 system at two sequencer companies (Macrogen, Korea/LAS Science, Korea).

### Single-cell RNA sequencing data processing

The Python pipeline available at https://github.com/indrops/indrops was used to read raw sequencing data with modified parameters (Bowtie 1.2.3 m = 200, n = 1, l = 15, e = 1,000) followed by alignment to the Ensembl mouse reference genome (GRCm38 release 102) to obtain a cell-gene expression matrix. In the R environment (version 4.3.0), the Seurat package (version 4.3) (Butler et al., 2018; Stuart et al., 2019; Hao et al., 2021) was used to filter cells, reduce dimensionality, cluster with Louvain, and project cells with UMAP. Cells with low quality, defined as having less than 1,000 reads and 500 UMI-filtered mapped reads, were prefiltered. Doublets were identified and removed using the ScDblFinder package (Germain et al., 2021). Using the SCTtransform wrapper, the G2M phase score, the S-phase score, the UMI count, and the proportion of mitochondrial genes were regressed out. Our samples were integrated with parts of a public dataset (GSE129788) as a reference based on L2 normalization using Harmony (Korsunsky et al., 2019) for improved data integration and cell-type identification. Initial clustering was used to group cells using the Louvain graph clustering algorithm, and cells were annotated using the topmost significant upregulated cluster-specific genes. Microglia were isolated and preferentially expressed markers of microglia or macrophages. The subset of microglia was processed through dimensionality reduction, Louvain clustering, and projection into UMAP. To examine changes in gene expression, we used the MAST (Yajima, 2023) test based on a log fold change threshold of 0.25 and an adjust *p*-value of *p* < 0.1 (about *p*-value <1 × 10^−5^) in Seurat. The weighted kernel density estimation was calculated and visualized using the Nebulosa package (Alquicira-Hernandez and Powell, 2021). The results of the differential gene expression were plotted as volcano plots using the EnhancedVolcano package (Lewis, 2023). GSEA was performed with the clusterProfiler package (Yu et al., 2012; Wu et al., 2021). We identified pathways with *p*-value <0.01 as significant pathways. The pathways we showed in the dot plot were plotted using the ggplot2 package (Wickham, 2016). Pseudotime analysis was performed using the Monocle3 package (Cao et al., 2019). A beginning point for the trajectory was selected manually based on the expression of canonical markers of homeostatic microglia. To analyze gene expression dynamics along the pseudotime, we used the tradeseq package (Van den Berge et al., 2020). An association test was used to determine gene expression changes along the pseudotime lineage. Next, we applied the patternTest function to evaluate the smoothed gene expression patterns along the pseudotime between the two principal trajectories. The scores of the three gene-sets previously reported were calculated by the UCell package (Andreatta and Carmona, 2021).

### Public dataset processing

We reanalyzed public datasets of single-cell transcriptome profiles from previously published research. The count data from the Gene Expression Omnibus (GEO) database with the accession numbers GSE121654, GSE127893, GSE129788, GSE165306, and GSE166548 datasets (Hammond et al., 2019; Sala Frigerio et al., 2019; Ximerakis et al., 2019; Grubman et al., 2021; Safaiyan et al., 2021) were downloaded. Subsequently, all datasets were integrated using Harmony, and the standard Seurat analysis workflows were implemented as previously described.

Information on the number of animals and the number of replicates for experiment

### Statistical analyses

The statistical analyses involved in the western blot analysis and the immunostaining analysis were conducted with GraphPad Prism 9 (GraphPad Software Inc.). For the statistical analysis of the transcriptome, R environments were employed, while for the proteome analysis, the PD 3.0 software was utilized. All data were presented as the mean ± standard error of the mean (SEM).

## Results

### Differential formation of axonal spheroids in various brain regions of 5xFAD mice

**FIGURE 1:**
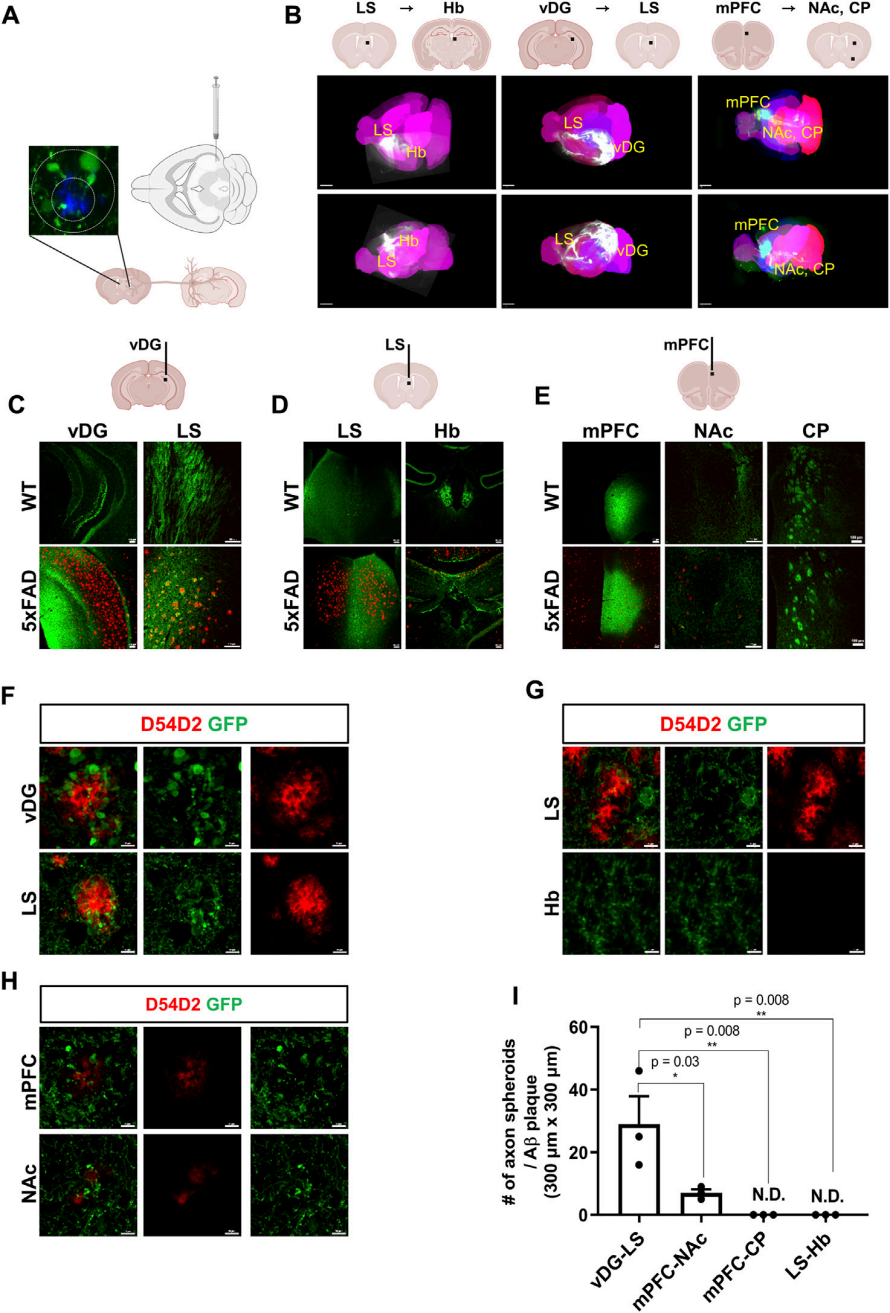
Visualization of differential axonal spheroid formation in 5xFAD mice using AAV tracers **(A)** A schematic drawing depicts the injection of AAV-GFP into the vDG to trace the axonal projection into the LS. **(B)** Whole-brain imaging exhibits each track of AAV injected into the vDG, the LS, and the mPFC (6-month-old, WT, n = 4, female). The representative neuronal projections of each injected site were visualized: LS to Hb (LS → Hb), vDG to LS (vDG → LS), and mPFC to the NAc and CP (mPFC→ NAc/CP) of mice. Each black bar in the schematic drawings indicates the AAV-injected position. Allen brain atlas was used as a reference for mouse three-dimensional images (pink). **(C)** Representative images of an AAV-injected vDG region and the axonal target region in the LS are presented. Aβ plaques were stained using anti- Aβ antibodies (D54D2, red), and AAV-EGFP tracers were stained for EGFP (6-month-old, n = 4, each group, female). **(D)** Representative images of the AAV-injected LS and its main axonal target in the Hb (6-month-old, n = 4, each group, female) **(E)** Representative images of the AAV-injected mPFC and its major axonal targets, such as the NAc and CP of WT and 5xFAD mice (6-month-old). Imaging of AAV-injected mice was conducted 3 weeks after stereotaxic injection (6-month-old, n = 4, each group, female). **(F∼H)** Representative images of axonal spheroids and Aβ plaques (D54D2, red) at the AAV-injected sites and the axonal target sites (F, vDG → LS; G, LS → Hb; H, mPFC→ NAc). **(I)** A plot depicts the number of axonal spheroids/Aβ plaque in each projection: vDG → LS, mPFC → NAc, mPFC → CP, and LS → Hb. Data represent means ± SEM, and *p*-values were calculated by an unpaired two-tailed *t*-test. **p < 0.01, *p < 0.05. Scale bars = 2 mm **(B)**; 100 µm **(C∼E)**; 10 µm **(F∼H)**.

Loss of synapses in AD patients is directly associated with cognitive decline (Selkoe, 2002; Barthet and Mulle, 2020). To investigate the regional selectivity of axonal dystrophies in an amyloidopathy mouse model, we used 5xFAD mice that carry mutations in the amyloid precursor protein (APP) and PSEN1 genes leading to Aβ accumulation to introduce AAV tracers expressing EGFP (AAV1-hSyn1-EGFP-P2A-EGFPf-WPRE-HGHpA) into the ventral dentate gyrus (vDG), the lateral septum (LS), and the medial prefrontal cortex (mPFC) (Figures 1A,B). These injections targeted the major axonal tracts of the vDG to the LS, the LS to the habenula (Hb), and the mPFC to the nucleus accumbens (NAc) or the caudate putamen (CP), respectively. To visualize three-dimensional axonal projection from each injection site, we took advantage of the brain clearing and three-dimensional rendering of EGFP tracers using the mouse brain expressing the AAV tracers. The dorsal and lateral brain-wide imaging showed major axonal projections targeting the LS, the Hb, and the subcortex (Figure 1B). The coronal sections of the AAV-expressing mouse brains were used to localize the EGFP signals in dystrophic axons adjacent to the Aβ plaques in 5xFAD mice compared to the normal axonal projections in WT mice. The strong EGFP signals of the axons originally injected in the vDG, the LS, and the mPFC were observed at the LS, the Hb, and NAc/CP, respectively, in both wild-type (WT) and 5xFAD mice (Figures 1C–E). While Aβ plaques were predominantly present in the vDG, the LS, and the mPFC of 5xFAD mice, the EGFP-expressing axons exhibited distinct signals of axonal spheroids near each plaque. The vDG-LS tracts showed the most drastic Aβ plaques (D54D2, red) and axonal spheroids (EGFP) signals (Figure 1F), whereas the LS-Hb tracts did not exhibit such axonal spheroid signals (Figure 1G). In the case of the mPFC-NAc/CP tracts, the NAc showed the signals of Aβ plaques with tiny axonal spheroids, and the CP did not exhibit any detectable signals (Figure 1H, CP not shown). We quantified the number of axon spheroids within each Aβ plaque in the terminal region of the axonal tracts and found that the LS of the vDG-LS tracts had a significantly higher number of axonal spheroids compared to the other tracts (Figure 1I). These results show that the vDG neurons projecting to the LS are susceptible to forming an axonal spheroid structure close to Aβ plaque.

### Inference analysis of single-cell RNA sequencing (scRNAseq) data predicts regional differences in cilium-related gene expression

**FIGURE 2:**
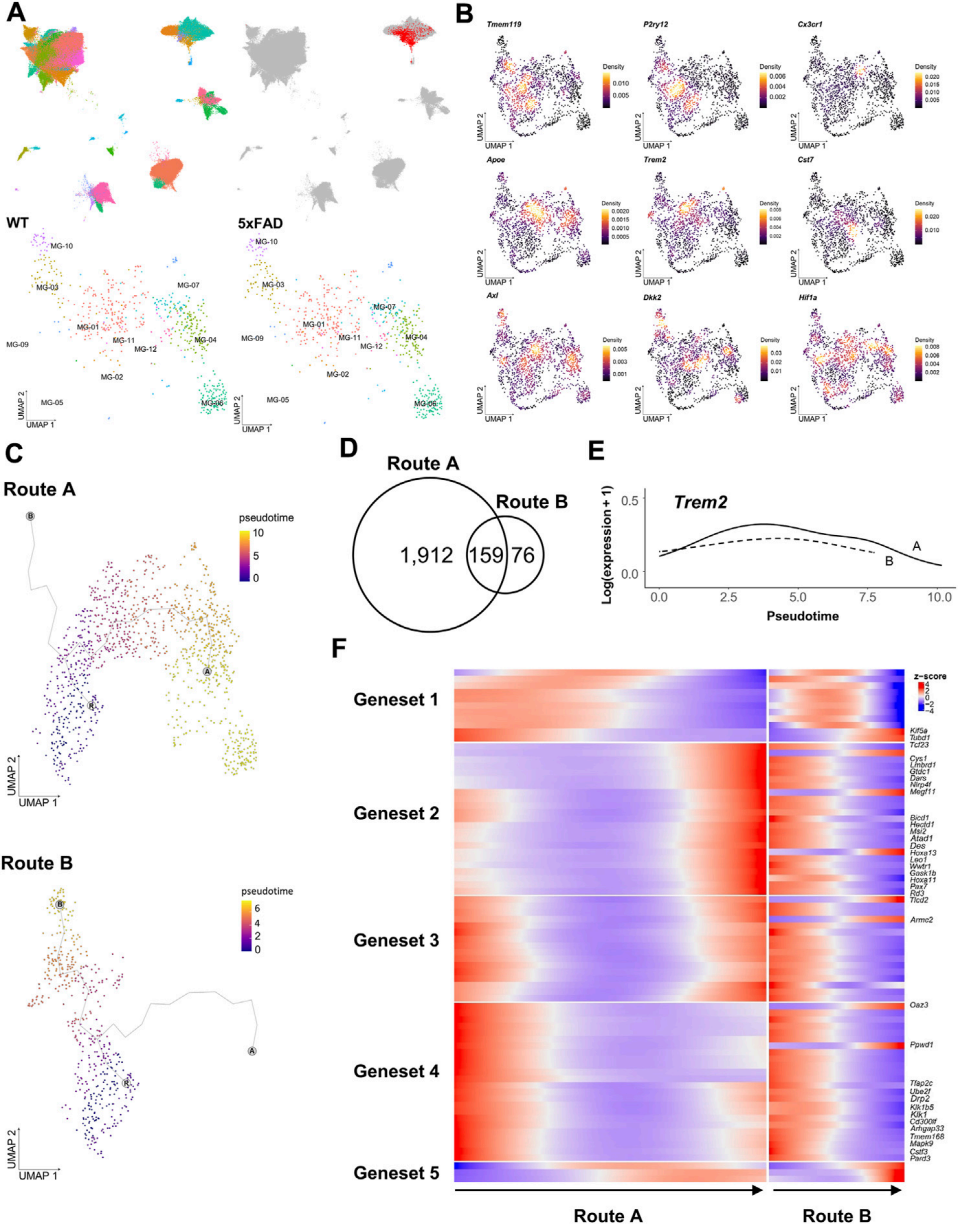
Differential trajectories of DAM from WT microglia were analyzed using the scRNAseq dataset. **(A)**, **(upper)** UMAPs depict 144,185 cells sequenced from 16 samples of the hippocampus (88,011 cells), 4 samples of the septum (20,952 cells), and 7 samples of the Hb (35,222 cells), colored by each cell type (left) and highlighting a microglia-specific cluster (right). **(A)**, **(lower)** UMAP depicts subclusters of 1,660 microglia selected from **(A)** representing each cell obtained from WT (366 cells at 3 months, 124 cells at 5 months, 26 cells at 6 months, 240 cells at 9 months, and 292 cells 12 months) and 5xFAD (201 cells at 3 months, 337 cells at 5 months, and 74 cells at 6 months) **(B)** Kernel density estimation with the Nebulosa package (Alquicira-Hernandez and Powell, 2021) shows the distribution of three homeostatic microglial feature genes (top) and six DAM feature genes (middle and bottom). **(C)** Trajectory plots showing the bifurcation of microglial progression into two principal trajectories (R: main root node, A: end node of Route A, B: end node of Route B, colored by pseudotime **(D)** Venn diagram of genes significantly associated with pseudotime in each trajectory **(E)** *Trem2* gene expression along pseudotime in Route A (solid line) and Route B (dotted line) **(F)** Heatmap showing five clustered expressions of genes significantly associated with pseudotime in Route A and Route B. The root was set by microglia in the MG-02 cell cluster of 3-month-old WT mice.

Microglia are phagocytic cells that engulf a variety of debris, including Aβ plaques, myelin fragments, apoptotic cells, and extracellular harmful substances. This diversity of phagocytic targets is reflected in the heterogeneous microglial subtypes, each with its own unique set of receptors and signaling pathways (Ribeiro Xavier et al., 2015; Krasemann et al., 2017; Butler et al., 2021; Clayton et al., 2021; Huang et al., 2021). As demonstrated in stereotaxic experiments, the vDG-septum tract of 5xFAD mice implies the presence of microglia that are associated with these axonal abnormal structures in the septum. To profile the regional heterogeneity of microglia, including the septum, we performed single-cell transcriptome profiling isolated from the hippocampus, the septum, and the Hb of WT and 5xFAD mice using an inDrop™ platform (Klein et al., 2015). We captured a total of 144,185 cells with 38,075 feature genes, from 27 independent libraries (Figure 2A). The cell types of 50 clusters were annotated by examining marker genes and comparing them to those in a previous study (Ximerakis et al., 2019). A subset of microglia among these clusters was identified with the most significantly upregulated genes (*Cst3*, *Ctss*, *C1qa*, *C1qb, B2m*, Supplementary Figure S1). A total of 1,660 microglia were projected to new UMAP dimensions, and clustered into the twelve microglia clusters that were named MG-01 to MG-12 (Figure 2A bottom).

The microglia in WT mice were primarily characterized as homeostatic, expressing genes encoding transmembrane protein 119 (*Tmem119*) (Satoh et al., 2016), P2Y purinoreceptor 12 (*P2ry12*), and CX3 chemokine receptor 1 (*Cx3cr1*) (Butovsky et al., 2014). The MG-02 cell cluster had dense expression of the *Tmem119* and *P2ry12* genes, indicating that it is a homeostatic microglia-like cell cluster (Figure 2B, Supplementary Figure S2A,B). Microglia from WT and 5xFAD mice overlapped across the seven microglia clusters. Of the seven identified clusters, the MG-01 cell cluster demonstrated more elevated expression of *Apoe* (Supplementary Figure S2C,D), along with *Trem2* and *Cst7* genes previously known as DAM genes (Keren-Shaul et al., 2017; Krasemann et al., 2017). These findings suggested that the MG-01 cluster was comprised of DAM. The distribution of *Axl* and *Dkk2* expression indicated that the MG-03 and the MG-10 cell clusters were identified as activated response microglia and/or a DAM cluster (Aghaizu et al., 2023). The MG-04, the MG-06, the MG-07, and the MG-09 cell clusters were distinguished by their expression of hypoxia-inducible factor-1α (*Hif-1a*). The MG-03 and the MG-10 cell clusters consisted primarily of microglia isolated from the hippocampal region, while the other four cell clusters, including the MG-01, consisted primarily of microglia isolated from the septum. This result suggests that there were regional differences in the microglial transcriptome and in the distribution of DAM. Interestingly, microglia from the septum, the hippocampus, and the Hb regions in the MG-01, the MG-04, the MG-07, and the MG-09 cell clusters overlapped regardless of age (Supplementary Figure S2A,B).

It is assumed that homeostatic microglia in the normal and healthy states progressively undergo a transition into DAM in a pathological condition via extracellular stimuli. The application of pseudotime analysis to the scRNAseq data has revealed the existence of five distinct trajectories with the microglia clusters. Two primary trajectories have been identified, originating from the MG-02 cell cluster and then bifurcating toward either the MG-01 (Route A) or the MG-03 cell cluster (Route B, Figure 2C). These trajectories were consistent with the spatial distribution of regional differences in DAM described earlier. To characterize the progress of hippocampus-enriched DAM (Route B) and septum-enriched DAM (Route A), we looked for the genes whose expression varied significantly over pseudotime within each trajectory. We found 1,912 genes in Route A, 76 genes in Route B, and 159 genes in both routes (Figure 2D). Given that both routes originated from a common MG-02 cell population, certain genes with different expression patterns may explain the characteristics of hippocampus-enriched DAM and septum-enriched DAM among the 159 genes found to be significantly associated with pseudotime. The *Trem2* gene, as a canonical marker of DAM, exhibited comparable trends of slight fluctuations over time in both primary trajectories (Figure 2E). This aligned with *Trem2* expression, which is initially increased in DAM. In contrast, it was observed that out of the 159 genes that were previously identified, 26 genes exhibited significantly different expression patterns in Route A and Route B (Figure 2F). The genes *Kif5a*, *Cys1*, and *Armc2* were commonly associated with ciliary assembly (Tao et al., 2009; Novas et al., 2018; Hesketh et al., 2022). The finding indicates that the variation in the expression of genes associated with cilia may be one of the contributing factors to the regional heterogeneity observed in both routes.

### Analysis of differential gene expression in WT microglia reveals that cilium-related gene expression is regionally regulated

Microglia isolated from WT and 5xFAD mice were similarly distributed across seven microglial clusters, as determined by UMAP dimensionality reduction analysis of the microglial subtypes. The findings suggested the presence of a microglial subtype in the brains of WT mice with gene expression features related to the DAM subtype identified in 5xFAD mice. To test whether the analysis of microglial single-cell transcriptome datasets supports these findings, the five publicly available datasets (Hammond et al., 2019; Sala Frigerio et al., 2019; Ximerakis et al., 2019; Grubman et al., 2021; Safaiyan et al., 2021) were acquired and integrated using the same method used in the previous analysis (Supplementary Figure S3A). This allowed us to identify a total of 43 clusters of microglia. To analyze marker gene expression in the microglial subclusters, we selected 9 clusters consisting of more than 1% microglia from 5xFAD mice and APP/PS1 mice (Supplementary Figure S3B). We further selected 10 clusters that had markers for *Spp1*, *Trem2*, *Axl*, *Lpl*, *Lgals3*, and *Gpnmb*, and 7 clusters that had markers for *Cx3cr1*, *P2ry12*, and *Tmem119*. This down-sampling process produced a total of seventeen clusters containing 36,013 cells (Supplementary Figure S3C). We examined the canonical marker gene expression in the microglial subclusters (Supplementary Figure S3D). This led to the identification of two microglial subtypes in the WT mouse brains that exhibited characteristics like DAM gene expression, supporting our previous findings using scRNAseq data from the septum.

**FIGURE 3:**
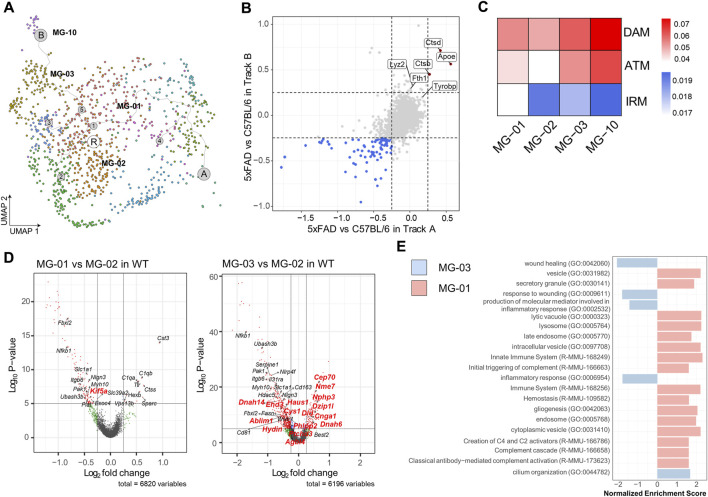
Differential regulation of genes regulating ciliogenesis and secretion in septum- and hippocampus-enriched microglia **(A)** MG-01 (belonging to Route A, septum enriched, upper), MG-03 (belonging to Route B, hippocampus enriched, bottom), MG-10 (belonging to Route B, hippocampus enriched) cell clusters in WT mice, colored by each subcluster **(B)** Four-way plots showing differential expression analysis between 5xFAD and WT mice in the cell population of Route A and the cell population of Route B. Red dots indicate a mean fold change higher than 0.25 and a *p*-value less than 0.05, whereas blue dots indicate a fold change less than 0.25 and a *p*-value less than 0.05. **(C)** Heatmap showing gene scores calculated by 14 DAM, 20 ATM, and 20 IRM feature genes. The columns in the heatmap represent the MG-01, the MG-02, the MG-03, and the MG-10 cell cluster. **(D)** Volcano plots depicting the differentially expressed genes observed in the comparison between MG-01 and MG-03 *versus* MG-02 in WT mice. Differentially expressed gene analysis yielded 8 upregulated genes and 99 downregulated genes in the MG-01, 109 upregulated genes and 413 downregulated genes in the MG-03 cell cluster (Genes marked with red dots have an absolute value of average fold change greater than 0.25 and an adjust *p*-value less than 0.1). Among these genes, those highlighted in red are the ones that overlapped with genes listed in CiliaCarta. **(E)** Bar graph showing GSEA based on the GO and the Reactome database for both MG-01 and MG-03 clusters. For the MG-01, GSEA identified 19 pathways based on the GO-CC, 16 pathways based on the GO-BP, 3 pathways based on the Reactome (*p*-value less than 0.01). For the MG-03, GSEA revealed 29 pathways based on the GO-CC and 38 pathways based on the GO-BP (*p*-value less than 0.01). The bar graph represents the results of a keyword search within these pathways, highlighting terms such as “wound,” “cilium,” “inflammatory,” “complement,” “hemostasis,” “secretory,” “vesicle,” “vacuole,” “endosome,” “lysosome,” and “exosome.”

Our previous scRNAseq analysis identified three microglial clusters in the mouse brain that exhibited comparable expression of DAM signature genes, such as MG-01 in Route A, MG-03, and MG-10 in Route B. These clusters all expressed DAM signature genes such as *Apoe*, *Dkk2*, and *Axl* (Figure 3A). To ascertain the similarity of DAM stages between the Route A and the Route B cell populations, we compared the gene expression profiles of these cell populations in WT and 5xFAD mice (Figure 3B). We found that both cell populations had two common DAM signature genes (*Apoe* and *Ctdb*) and three common axon tract microglia (ATM) signature genes (*Apoe*, *Ctdb*, and *Ctds*), which were significantly upregulated in 5xFAD mice. The Route B cell populations, which are predominantly hippocampus-derived microglia, had three DAM signature genes (*Ftl1*, *Lyz2*, and *Fth1*) that were significantly upregulated in 5xFAD mice. To determine if there were microglial subtypes in the WT mouse brain that exhibited DAM features in our data containing a regional distribution, we examined microglia derived from WT brains. Gene score values were calculated using known 14 DAM genes (*Ank, Apoe, Axl, Ccl6, Cd63, Cd9, Csf1, Cst7, Ctsz, Igf1, Itgax, Lpl, Spp1, Tyrobp*), 20 axon tract microglia (ATM) genes (*Spp1, Gpnmb, Igf1, Lgals3, Cd9, Fabp5, Lpl, Syngr1, Pld3, Ctsl, Lgals1, Lilrb4a, Ccl9, Anxa5, Gm1673, Csf1, Cd63, Gm10116, Anxa2, Apoe*), and 20 interferon-responsive microglia (IRM) signature genes (*Ccrl2, Csprs, Dhx58, Ifit1, Ifit2, Ifit3, Ifit3b, Ifitm2, Ifitm3, Irf7, Isg15, Isg20, Ly6a, Oas1a, Oas1g, Oas3, Oasl1, Oasl2, Stat2, Usp18*) (Figure 2C) (Hammond et al., 2019; Lall et al., 2021). The MG-02 cell cluster enriched in WT mice, which was considered the homeostatic microglia, had relatively low gene scores for DAM and ATM. The MG-01 cell cluster from WT mice, with a high proportion of septum-derived microglia, maintained high gene score values for DAM and ATM but had the lowest values for IRM. In contrast, the MG-03 cell cluster from WT mice, which was dominated by microglia from the hippocampus, had relatively high gene score values for DAM and ATM and the highest value for IRM. Similarly, the MG-10 cell cluster from WT mice showed high gene score values for DAM, ATM, and IRM. This may imply that WT microglia are regionally heterogeneous and transcriptionally primed to respond closely to the pathological needs of the cells comprising the tissues.

Subsequent differential expression gene analysis was conducted on the MG-01 and the MG-03 in comparison to the MG-02 in WT mice (Figure 3D, Supplementary File S1). The analysis yielded a list of genes associated with cilia that exhibited significant alterations in expression (with adjust *p*-value <0.1 and an average absolute Log_2_ Fold change >0.25) based on data from CiliaCarta (van Dam et al., 2019). Only the *Kif5a* gene among those cilia-related genes exhibited downregulation in differential expression analysis between the MG-01 and MG-02 cell clusters in wild-type mice. However, it was discovered that 14 cilia-related genes overlapped with the differentially expressed genes in the MG-03 cell cluster in WT mice as compared to the MG-02 cell cluster, indicating the presence of ciliary dysfunction. To gain insights into the differential enrichment of signal pathways among regional microglial subtypes, we performed gene-set enrichment analysis (GSEA) using the differentially expressed genes. GSEA, based on the Gene Ontology (GO) and the Reactome databases, revealed a notable increase in genes related to cilium organization within the MG-03 cell cluster (Figure 3E, Supplementary File S1). On the other hand, the cluster of MG-01 cells displayed an upregulation of diverse vesicle-related genes such as secretory granules, lytic vacuole, and cytoplasmic vesicle. Additionally, we observed a decrease in genes related to immune system, lysosome, and endosome within the MG-01 cell cluster. These results suggest that the regionally heterogeneous microglial subtypes may be involved in distinct stages or priming status as determined by cilium-, vesicle-, and secretion-related genes.

### Dampening of the primary cilia in microglia in amyloidopathy

**FIGURE 4:**
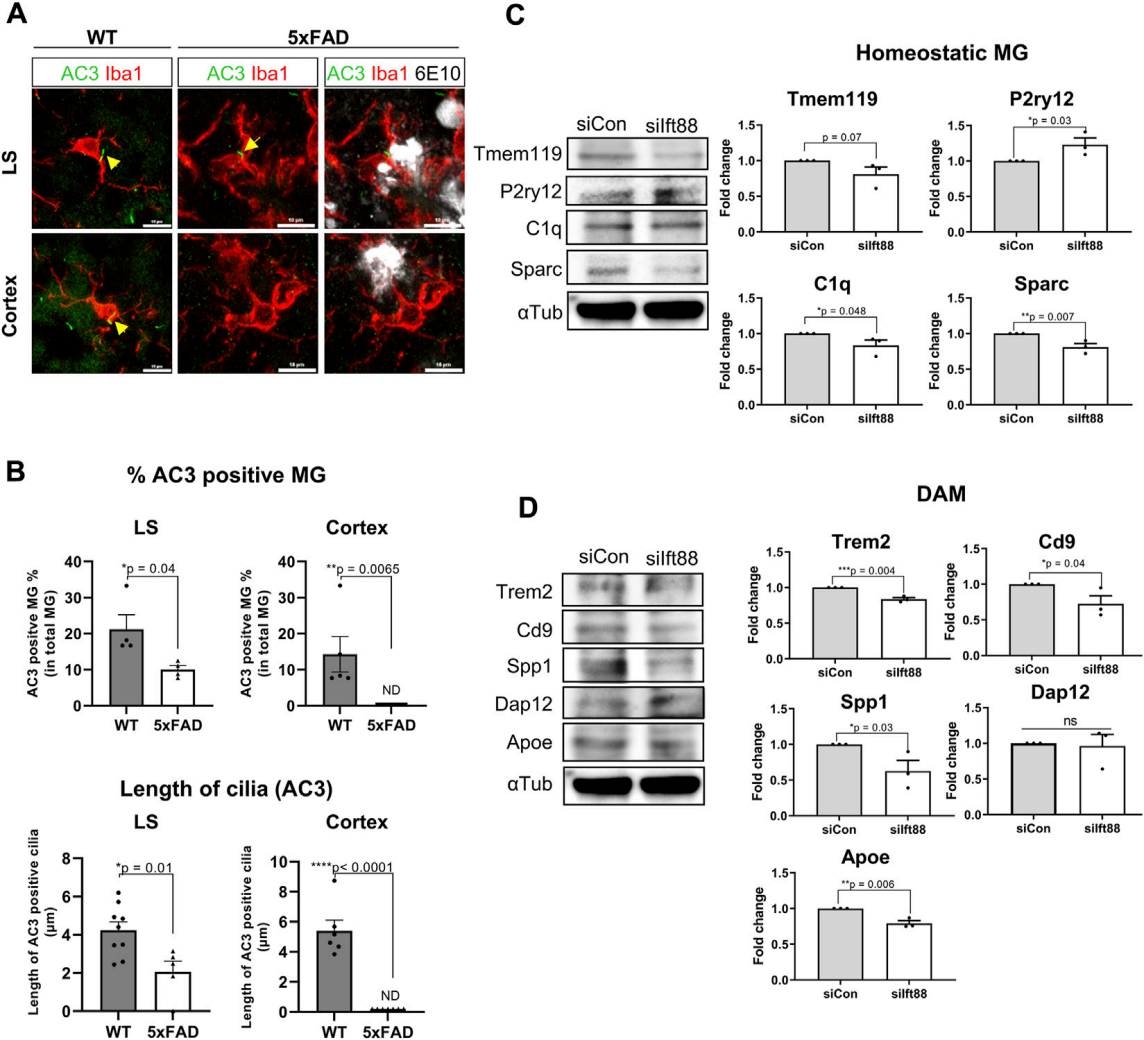
Repression of microglial primary cilia in 5xFAD mice **(A)** Representative images of AC3 (green)-positive microglia (MG) stained for Iba1 (red) in the LS and the cortex of WT and 5xFAD mice (6-month-old). In 5xFAD mice, Aβ plaques were stained using 6E10 antibodies (white). **(B)** Plots depict the percentage of AC3-positive microglia and the length of AC3-stained primary cilia, respectively. Images were taken from the LS (1024 ㎛^2^) and the cortex (1024 ㎛^2^) of WT and 5xFAD mice (5-month-old, n = 4, each group, male) **(C)** Western blot analysis shows the differential expression of the homeostatic microglial markers in BV2 cells, which were harvested 24 h after transfection with siIft88. Plots represent protein expression normalized by α-tubulin (αTub, n = 3). **(D)** Western blot analysis shows the differential expression of the DAM markers in BV2 cells transfected with siIft88 for 24 h. Plots represent protein expression normalized by αTub (n = 3). **(B–D)** Data represent means ± SEM, and *p*-values were calculated by an unpaired two-tailed *t*-test. ****p < 0.0001, ***p < 0.001, **p < 0.01, *p < 0.05, ns (non-significant). Scale bars = 10 µm.

The previous scRNAseq analysis of microglial subclusters in different brain regions revealed that cilium-related genes may regulate the status of microglia. To examine the role of the microglial primary cilia in a pathological condition such as amyloidopathy, we compared the expression of adenylate cyclase type III (AC3), a well-established cilia marker found in primary cilia throughout the brain (Bishop et al., 2007), in microglia from 6-month-old WT and 5xFAD mice in the LS and the cortex. We identified AC3-labeled primary cilia localized in the region between the cell body and the major process in microglia expressing Iba1. In the LS, both WT and 5xFAD mice showed microglia with primary cilia, regardless of Aβ plaques. However, in the cortex of 5xFAD mice, AC3 was not detected in microglia (Figures 4A,B). The percentage of AC3-positive microglia in the LS and the cortex was significantly lower in 5xFAD mice compared to WT mice. Furthermore, microglia in 5xFAD mice exhibited shorter primary cilia in the same brain region (Figure 4B). Since the primary cilia face outside cells, the protruding structure could be damaged by extracellular toxic substances such as Aβ. These results indicate microglia in the cortex are more likely to be affected in their primary cilia by extracellular amyloidopathy. To examine the involvement of primary cilia in microglial status, we used BV2 cells, a mouse microglial cell line, to downregulate the expression of Ift88 using siRNA transfection. Subsequently, we examined protein markers associated with both homeostatic and activated microglial states. The knockdown of Ift88 led to a decrease in the expression of homeostatic microglial cell markers, such as Tmem119, C1q, and Sparc, except for P2ry12, which exhibited a significant induction (Figure 4C). Additionally, the expression of protein markers associated with activated microglial cells, including Trem2, CD9, Spp1, and Apoe, was reduced upon siIft88 treatment (Figure 4D). These findings suggest that Ift88 or primary cilia may be involved in triggering or maintaining the activated status of microglia such as DAM.

### Regulation of microglial secretion by primary cilia

**FIGURE 5:**
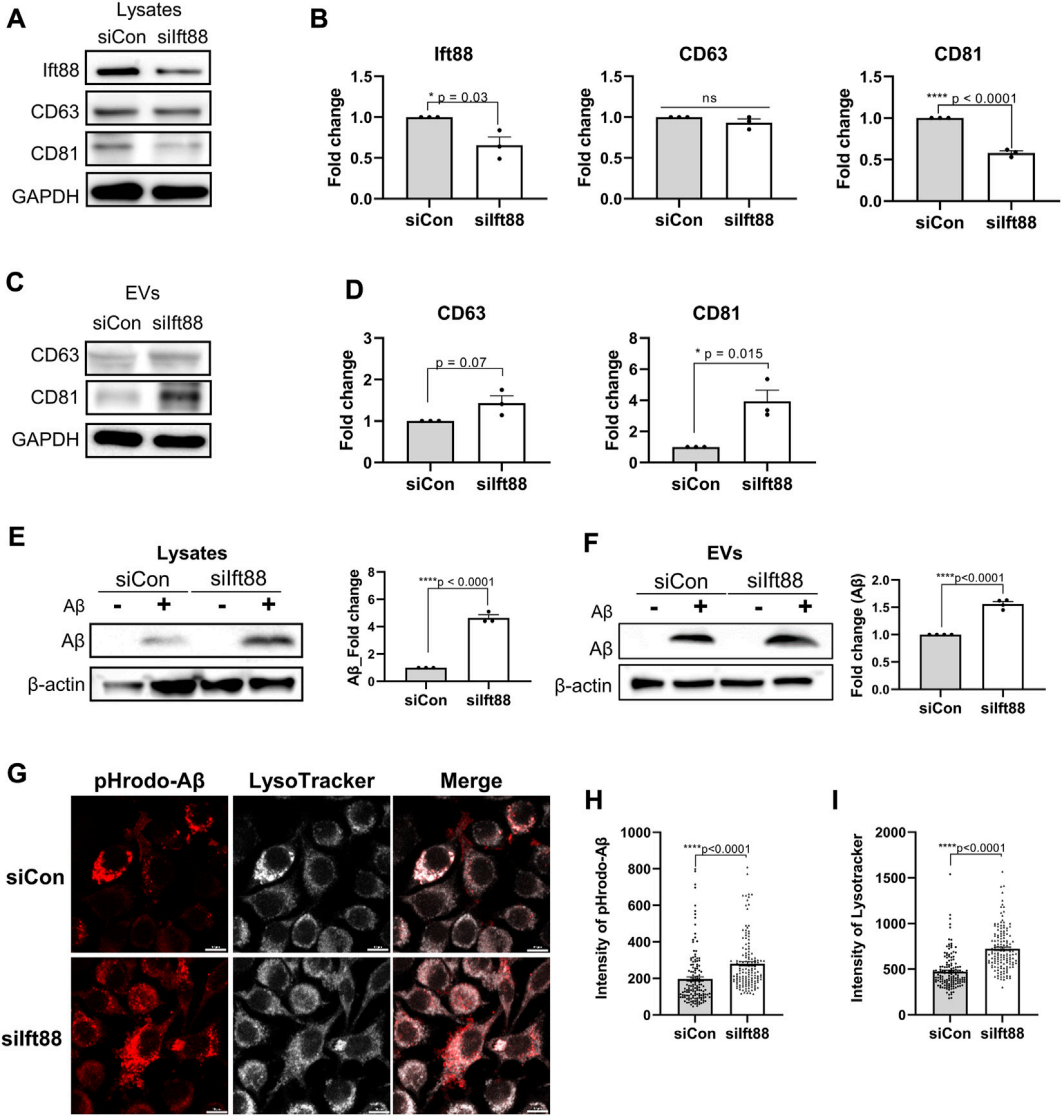
Promotion of phagocytosis and EV secretion in BV2 cells by silencing of Ift88 **(A)** A representative western blot result of Ift88, CD63, and CD81, analyzed using lysates of BV2 cells transfected with siCon or siIft88. **(B)** Plots depict the expression of Ift88, CD63, and CD81 from western blots in **(A)** (n = 3). **(C)** A representative western blot result of CD63 and CD81, analyzed using EVs isolated from the conditioned medium of BV2 cells transfected with siCon or siIft88. **(D)** Plots depict the expression of CD63 and CD81 from western blots in **(C)** (n = 3). **(E)** Western blot analysis of Aβ uptake, analyzed using lysates of BV2 cells transfected with siCon or siIft88, followed by treatment with 1 μM Aβ for 18 h. A plot depicts Aβ in cell lysates (n = 3). **(F)** Western blot analysis of Aβ secretion in EVs, analyzed using the EVs collected from the conditioned medium of the BV2 cells transfected with siCon or siIft88, followed by treatment with 1 μM Aβ for 18 h. A plot depicts the Aβ obtained in EVs (n = 4). **(G)** Representative images of Aβ phagocytosis and lysosome activity in BV2 cells after Ift88 inhibition. BV2 cells were transfected with siCon or siIft88, followed by treatment with 1 µM pHrodo-Aβ for 18 h. Fixed cells were stained for pHrodo-Aβ and LysoTracker to visualize the phagocytosis activity and lysosome activity, respectively. **(H), (I)** Plots depict the relative intensity of pHrodo-Aβ **(H)** and LysoTracker **(I)** (n = 4). Data represent means ± SEM, and *p*-values were calculated by an unpaired two-tailed *t*-test. ****p < 0.0001, ***p < 0.001, **p < 0.01, *p < 0.05, ns (non-significant). Scale bars = 10 µm.

Microglia in the DAM state exhibit upregulation of genes involved in EV secretion as well as phagocytosis of Aβ and dystrophic neurites. To assess whether the primary cilia of microglia regulate the biogenesis and secretion of EVs, we evaluated the protein expression levels of CD63 and CD81, a member of the tetraspanin family known to be specifically localized in EVs and crucial for EV biogenesis and secretion (Berditchevski and Odintsova, 2007). In BV2 cells transfected with siIft88, the levels of CD81 and Ift88 were significantly reduced in the cellular lysate. However, the expression of CD63 remained unchanged upon decreased Ift88 in BV2 cells (Figures 5A,B). Indeed, it is intriguing to note that upon the silencing of Ift88, a discernible elevation in CD63 expression was observed, accompanied by a statistically significant augmentation in CD81 levels (Figure 5C,D). These findings suggest that primary cilia may be involved in EV secretion and EV biogenesis in microglia. EV secretion can be influenced by lysosomal overload, and the removal of toxic substrates may alleviate cellular damage (Eitan et al., 2016; Hessvik and Llorente, 2018). To investigate microglial phagocytosis and lysosomal clearance, BV2 cells were treated with Aβ (1 µM) following transfection with either scrambled siRNA or siIft88. Remarkably, Aβ uptake was significantly augmented in BV2 cells transfected with siIft88 (Figure 5E). Furthermore, the release of Aβ within EVs was also enhanced (Figure 5F). To assess lysosomal activity in siRNA-transfected BV2 cells, we employed pH-sensitive dye-conjugated Aβ (pHrodo-Aβ) and LysoTracker. Silencing Ift88 in BV2 cells resulted in a greater proportion of cells positive for pHrodo-Aβ and LysoTracker compared to controls (Figure 5G). Moreover, the intensity of pHrodo-Aβ and LysoTracker signals was significantly increased in BV2 cells transfected with siIft88 (Figure 5H, I). These findings suggest that Ift88 deficiency may induce aberrantly heightened lysosomal activity while promoting the secretion of EVs containing Aβ in microglia.

### Silencing Ift88 in microglia alters EV proteomic profiles in response to exposure to Aβ

**FIGURE 6:**
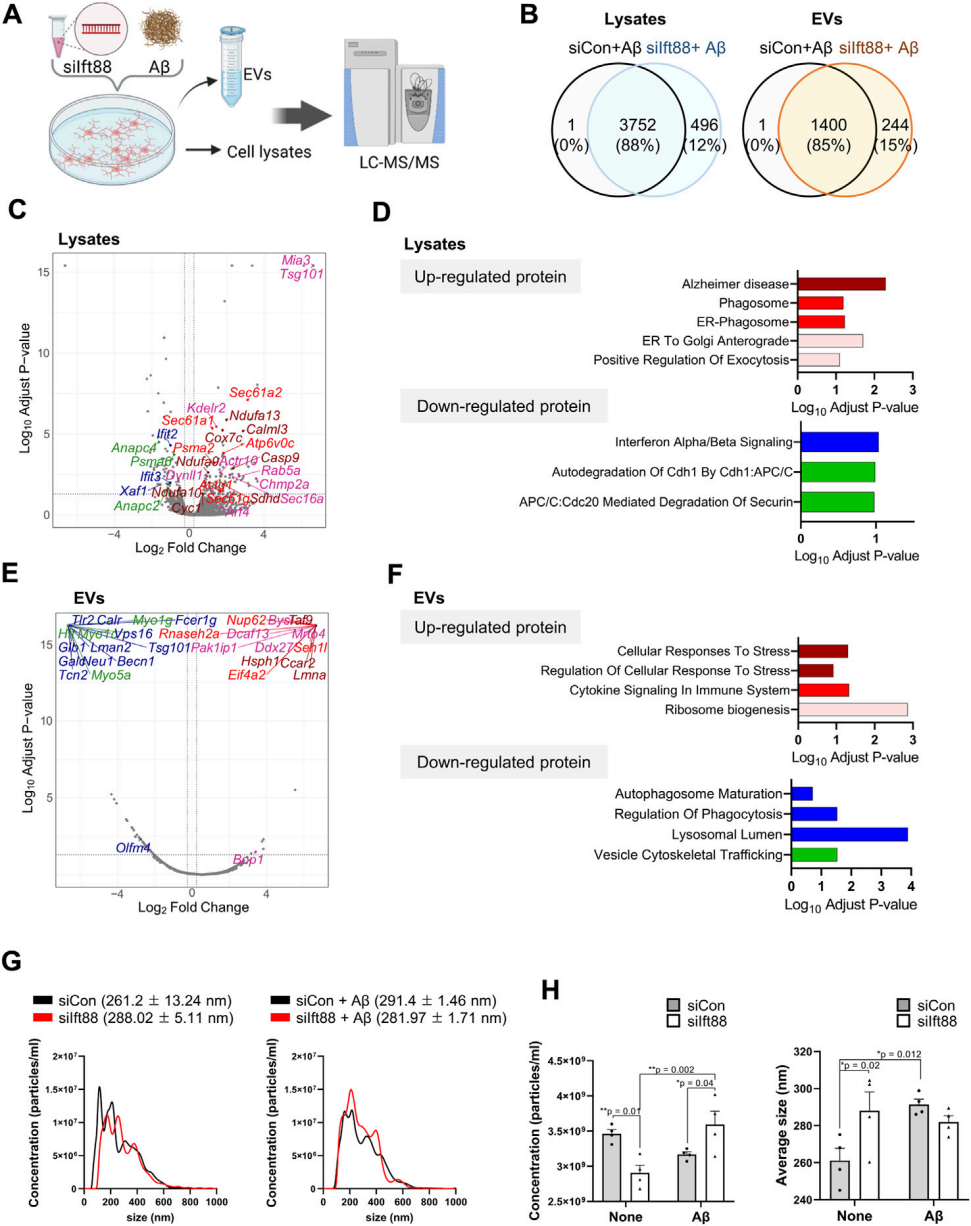
Proteomic analysis of cell lysates and EVs of BV2 cells transfected with siIft88 followed by treatment with Aβ **(A)** An experimental scheme shows proteomic analysis of cell lysates (n = 4, each group) and EVs (n = 4, each group) collected from the culture medium of BV2 cells after Ift88 inhibition combined with Aβ treatment. **(B)** Venn diagram indicating the number of identified proteins in BV2 cell lysates and EVs. Samples were obtained from BV2 cells after transfection with siCon or siIft88 followed by treatment with Aβ. **(C)** The volcano plot depicts the differentially expressed proteins in siIft8-transfected BV2 cell lysates by Aβ treatment. The cut-off values were determined by adjust *p*-value <0.05, and log2 (fold change) ≥ 0.25 (upregulated) or log2 (fold change) ≤ −0.25 (downregulated). **(D)** Up- and downregulated proteins were analyzed for enriched pathways using Enrichr (https://maayanlab.cloud/Enrichr/). Significantly enriched clusters (*p*-value <0.01) for up- and downregulated proteins are indicated in each graph. Representative proteins in each term of the ontology were marked with the same color in the volcano plot. **(E)** The volcano plot depicts the differentially expressed proteins in siIft8-transfected BV2 cells’ EVs by Aβ treatment. The cut-off values were determined by adjust *p*-value <0.05, and log2 (fold change) ≥ 0.25 (upregulated) or log2 (fold change) ≤ −0.25 (downregulated). **(F)** Up- and downregulated proteins were analyzed for enriched pathways using Enrichr. Significantly enriched clusters (*p*-value <0.01) for up- and downregulated proteins are indicated in each graph. Representative proteins in each term of the ontology were marked with the same color in the volcano plot. **(G)** Representative histograms of EV size distributions collected from BV2 cells transfected with siCon or siIft88 followed by treatment with or without Aβ (1 µM). The average size of EVs is presented above each histogram. **(H)** Statistical analysis of concentration (particles/ml) and average size of EVs isolated from the conditioned medium of BV2 cells, transfected with siCon or siIft88, and treated with or without Aβ (1 µM). Data represent means ± SEM, and *p*-values were calculated by two-way analysis of variance (ANOVA) followed by Turkey’s multiple comparison test. **p < 0.01, *p < 0.05.

To characterize the proteomic changes in microglia in response to Aβ treatment, we performed proteomic analysis of BV2 cells with the combination of Ift88 inhibition and treatment with Aβ. Figure 6A illustrates the experimental workflow utilized for proteomic analysis via LC-MS. Most proteins were identified in both lysates (88%) and EVs (85%) between the (siCon + Aβ) group and the (siIft88 + Aβ) group (Figure 6B). In the lysates of BV2 cells transfected with siIft88 and treated with Aβ, we observed 168 upregulated proteins (log2 fold-change ≥0.25, adjust *p*-value <0.05) and 126 downregulated proteins (log2 fold-change ≤ −0.25, adjust *p*-value <0.05) (Figure 6C). To gain insight into the functional implications of the differentially expressed proteins, we performed GSEA, which revealed significant alterations in various biological processes. The upregulated proteins were enriched in terms associated with Alzheimer disease, phagosome, ER-phagosome, ER-to Golgi anterograde, positive regulation of exocytosis, among others. Conversely, the downregulated proteins were enriched in terms such as interferon α/β signaling, autodegradation of Cdh1 by Cbh1:APC/C, APC/C:Cdc20 mediated degradation of Securin, and more (Figure 6D, Supplementary File S2). EV marker proteins such as Tsg101 were induced by Ift88, indicating the involvement of the primary cilia in the regulation of EV biogenesis and secretion. Overall, the enrichment analysis results suggest that the loss of Ift88 may induce an AD-associated state in microglia, stimulating secretion and decreasing phagocytic vesicle function. In the EVs of BV2 cells transfected with siIft88 and treated with Aβ, we observed that 76 proteins were upregulated (log2 fold-change ≥0.25, adjust *p*-value <0.05), while 196 proteins were downregulated (log2 fold-change ≤ −0.25, adjust *p*-value <0.05) (Figure 6E). The upregulated proteins were predominantly associated with cellular response to stress, regulation of cellular response to stress, cytokine signaling in immune system, and ribosome biogenesis. On the other hand, the downregulated proteins were enriched in terms related to autophagosome maturation, regulation of phagocytosis, lysosomal lumen, and vesicle cytoskeletal trafficking (Supplementary File S2). Representative proteins were depicted on the volcano plot, using distinct colors to denote their association with specific processes, such as dark red for cellular response to stress, bright red for cytokine signaling in immune system, green for vesicle cytoskeletal trafficking, and blue for autophagosome maturation, regulation of phagocytosis, and lysosomal lumen (Figure 6E, F). These findings suggest that the loss of Ift88 may induce proteomic alterations in EVs secreted by microglia, characterized by an increase in cellular response to stress and cytokine signaling in immune system and a decrease in autophagosome maturation and vesicle cytoskeletal trafficking. To validate the increase in vesicles, because of positive regulation of exocytosis, we performed nanoparticle tracking analysis (NTA) on EVs derived from BV2 cells transfected with siIft88 and treated with Aβ. Figure 6G illustrates the size distribution of EVs with and without Aβ treatment. The concentration of EVs was significantly increased in BV2 cells transfected with siIft88 and treated with Aβ, supporting the previous proteomic analysis (Figure 6H).

**FIGURE 7:**
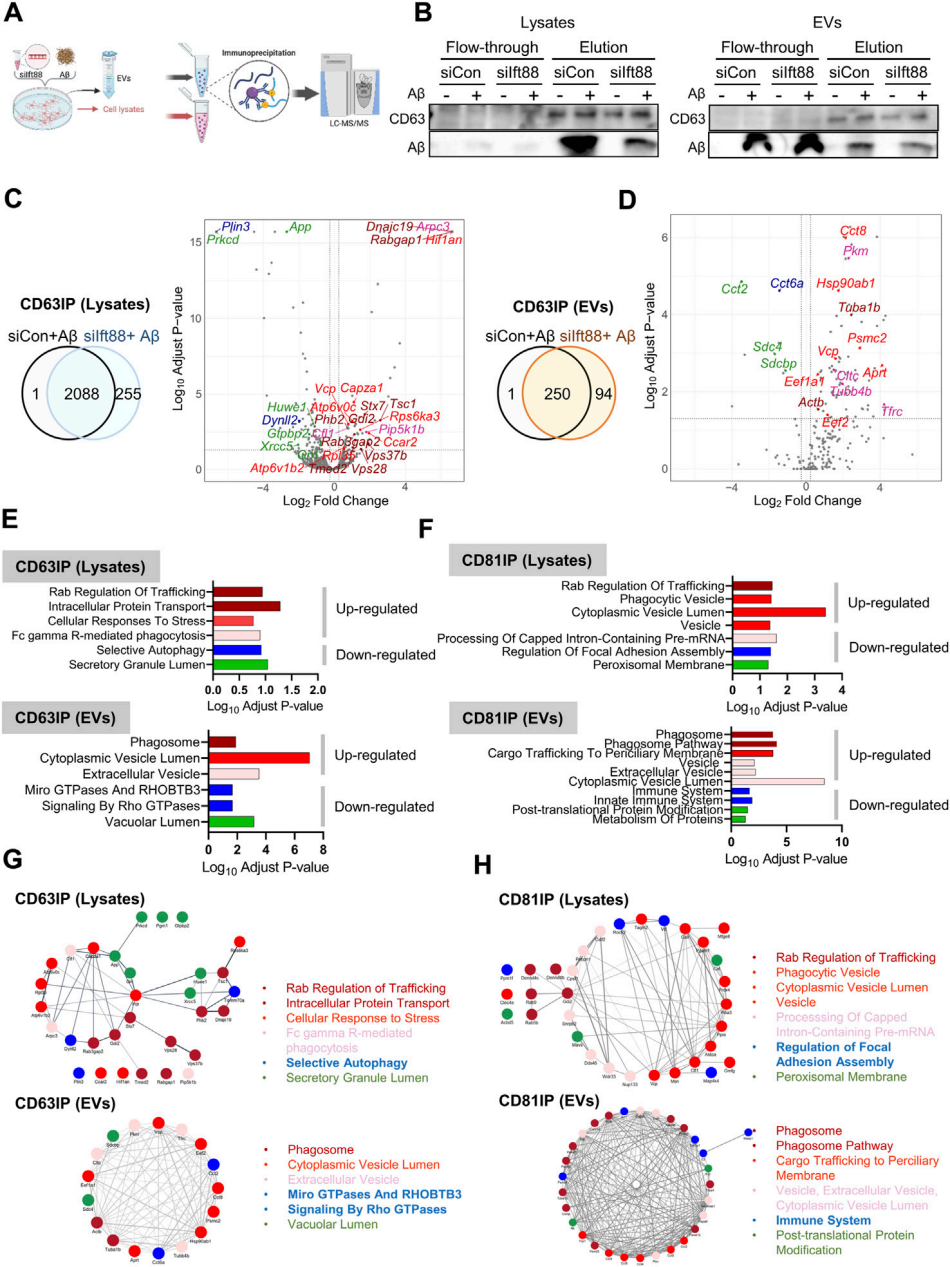
Proteomic analysis of CD63 or CD81-associated proteins in cell lysates (n = 4, each group) and EVs (n = 4, each group) of BV2 cells transfected with siIft88 in combination with Aβ **(A)** An experimental scheme describes the identification of CD63 or CD81 binding proteins by LCMS. **(B)** Western blot analysis of CD63 IP using cell lysates and EVs shows binding of Aβ with CD63 using cell lysates or EVs. CD63 IP using EVs shows CD63 EVs contain Aβ and other EVs, as revealed by Aβ in the flow-through fraction. **(C), D)** Venn diagram and volcano plot of CD63-binding proteins in cell lysates **(C)** and EVs **(D)** show identified proteins in upregulated proteins (adjust *p*-value <0.05, log2 (fold change) ≥ 0.25) or downregulated proteins (adjust *p*-value <0.05, log2 (fold change) ≤ −0.25) from BV cells, which were transfected with siIft88 followed by treatment with Aβ (1 µM), compared with BV2 cells transfected with siCon and treated with Aβ (1 µM). **(E, F)** GO pathway enrichment analysis of CD63-binding proteins **(E)** and CD81-binding proteins **(F)**. Up- and downregulated proteins were analyzed separately using the Enrichr. Significantly enriched clusters (*p*-value <0.01) for up- and downregulated proteins are indicated in each graph. Representative proteins for each GO term were color-coded and marked in the volcano plot as well. **(G, H)** PPI network analysis using the STRING database reveals a network among the selected GO enrichment pathways of CD63-binding proteins **(G)** and CD81-binding proteins **(H)** in the cell lysates and EVs.

EVs exhibit heterogeneity, and their proteomic contents provide insights into the signaling cascade mediated by these vesicles and the alterations in extracellular protein homeostasis, also known as, extracellular proteostasis (Geraghty et al., 2021; Krause et al., 2022). As demonstrated in previous findings (Figures 5B,D), the loss of Ift88 resulted in a decrease in CD81 levels in the lysate but an increase in CD63 and CD81 levels. To investigate the specific proteomic alterations in EVs associated with CD63 and CD81, we conducted a proteomic characterization of CD63-or CD81-decorated EVs in the lysate and EVs secreted from BV2 cells transfected with siIft88 and treated with Aβ, utilizing co-immunoprecipitation (Co-IP) with anti-CD63 or anti-CD81 antibodies. Figure 7A illustrates the experimental workflow for analyzing CD63-and CD81-binding proteins through liquid-chromatography mass-spectrometry (LCMS). Representative results of the Co-IP experiment showed that CD63 was enriched in the elution of the CD63-Co-IP, both in the lysates and EVs, and Aβ was also detected in the CD63-binding proteins (Figure 7B), indicating a potential interaction between Aβ and CD63 in BV2 cells and their secreted EVs. Most CD63-binding proteins were identified in both the lysates (89%) and EVs (72%) when comparing the (siCon + Aβ) group with the (siIft88 + Aβ) group (Figures 7C,D). In the lysates of BV2 cells transfected with siIft88 and treated with Aβ, 96 proteins were upregulated (log2 fold-change ≥0.25, adjust *p*-value <0.05), while 127 proteins were downregulated (log2 fold-change ≤ −0.25, adjust *p*-value <0.05) among the CD63-binding proteins (Figure 7C). In EVs, 75 proteins were identified as upregulated proteins (log2 fold-change ≥0.25, adjust *p*-value <0.05), and 8 proteins were identified as downregulated proteins (log2 fold-change ≤ −0.25, adjust *p*-value <0.05) among the CD63-binding proteins (Figure 7D). In the lysates, the upregulated proteins associated with CD63 were involved in processes such as Rab regulation of trafficking, intracellular protein transport, cellular response to stress, and Fc gamma R-mediated phagocytosis. The downregulated proteins associated with CD63 were related to selective autophagy and secretory granule lumen (Figure 7E, Supplementary File S3). In EVs, the upregulated proteins associated with CD63 were linked to phagosome, cytoplasmic vesicle lumen, and extracellular vesicle. The downregulated proteins associated with CD63 were involved in micro GTPase and RHOBTB3, signaling by Rho GTPase and vacuolar lumen (Figure 7E, Supplementary File S3). In the lysates of BV2 cells transfected with siIft88 and treated with Aβ, we observed that 147 proteins were upregulated (log2 fold-change ≥0.25, *p*-value <0.05 adjust *p*-value <0.05), while 113 proteins were downregulated (log2 fold-change ≤ −0.25, adjust *p*-value <0.05) among the CD81 binding proteins. Furthermore, in EVs, 76 proteins were identified as upregulated (log2 fold-change ≥0.25, adjust *p*-value <0.05), and 13 proteins were identified as downregulated (log2 fold-change ≤ −0.25, adjust *p*-value <0.05). Regarding the CD81-binding proteins in the lysates, the upregulated proteins were associated with Rab regulation of trafficking, phagocytosis vesicle, and cytoplasmic vesicle lumen, vesicle. Conversely, the downregulated proteins among the CD81-binding proteins were involved in processing of capped intron -containing pre-mRNA, regulation of focal adhesion assembly, and peroxisomal membrane (Figure 7F, Supplementary File S4). On the volcano plots, representative proteins were marked with distinct colors, such as dark red for AD, bright red for the secretory granule lumen, pink for the interleukin-12-mediated signaling pathway, green for focal adhesion, and blue for mRNA processing (Supplementary Figure S4A). In the EVs, the upregulated proteins associated with CD81 were linked to phagosome, phagosome pathway, cargo trafficking to periciliary membrane, vesicle, extracellular vesicle, and cytoplasmic vesicle lumen. The downregulated proteins among the CD81-binding proteins were associated with immune system, post-translational protein modification, and metabolism of proteins (Figure 7F, Supplementary Materail S4). On the volcano plot, representative proteins were marked with distinct colors, such as dark red for phagosome, bright red for cargo trafficking to periciliary membrane, faint red for vesicle, green for the metabolism of proteins and post-translation protein modification, and blue for immune system (Supplementary Figure S4B). Figure 7G,H summarize the protein-protein interaction (PPI) network among the up- or downregulated proteins of CD63 or CD81 binding proteins in each cell lysate and EV. These findings suggest that the loss of Ift88 may lead to an upregulation of stress response-related proteome in the extracellular space via EVs altering the extracellular proteostasis. The changes in the EV proteome and extracellular proteomic composition may affect the amyloidopathy in AD.

### The effect of the conditional inhibition of the primary cilia in microglia on the pathogenesis of AD

**FIGURE 8:**
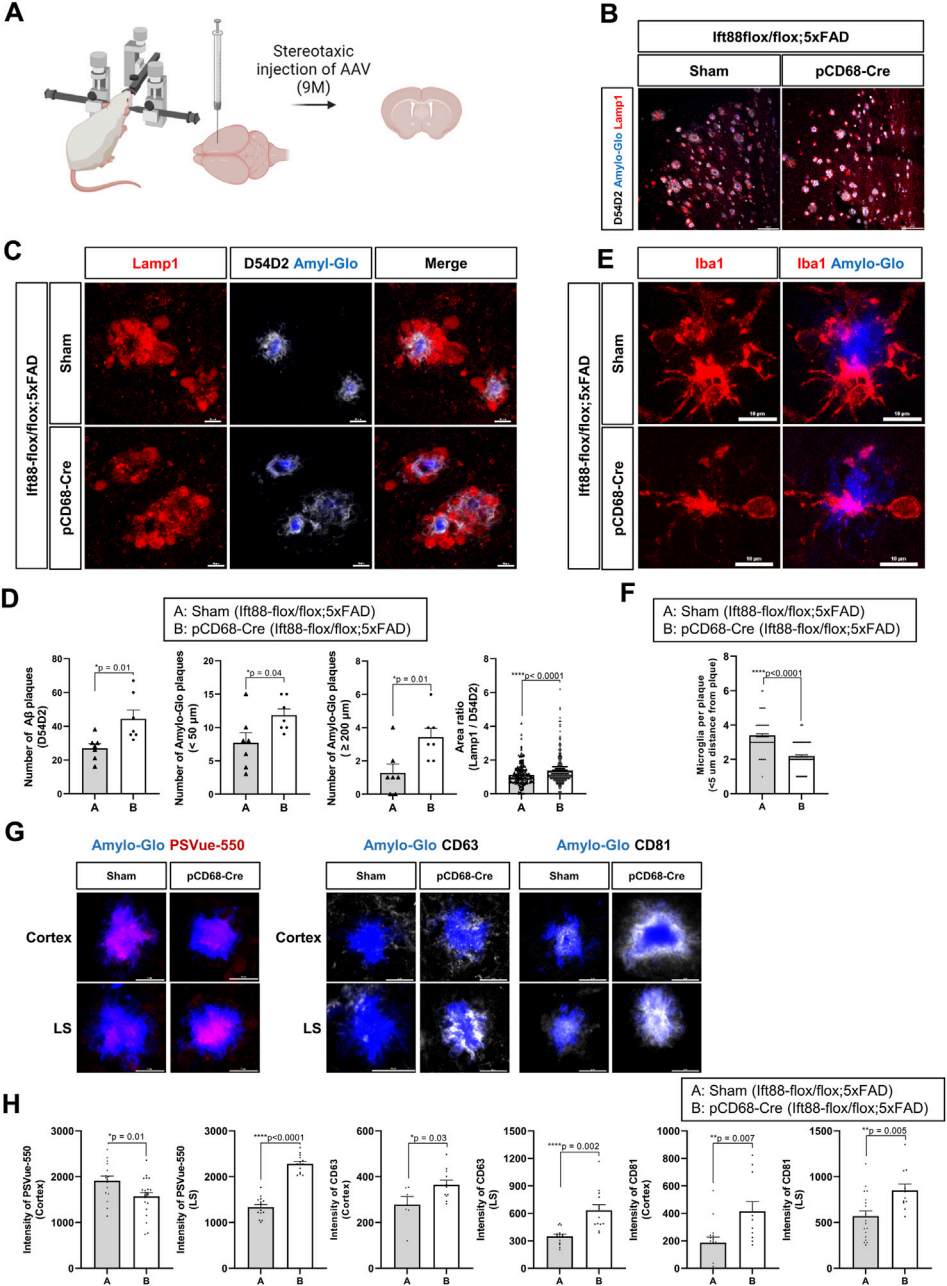
Increased Aβ plaques and dystrophic neurites in the LS of Ift88-flox/flox; 5xFAD (10-month-old) 1 month after intracerebroventricular (ICV) administration of AAV-pCD68-Cre **(A)** A schematic drawing illustrates the stereotaxic injection of AAV-pCD68-Cre into the lateral ventricle of Ift88-flox/flox; 5xFAD mice and the examination of the septal region after 1 month. **(B)** Representative low magnification images of the LS for Aβ plaques (D54D2, white), cored Aβ plaques (Amylo-Glo, blue), and dystrophic neurites (Lamp1, red) in 5xFAD (sham) (10-month-old, n = 3, one male and two females) and Ift88-flox/flox; 5xFAD (10-month-old, n = 3, one male and two females), injected with AAV-pCD68-Cre. **(C)** Representative images of dystrophic neurites (Lamp1, red) adjacent to Aβ plaques (D54D2, white). The cored Aβ plaques were counter-stained using Amylo-Glo (blue). **(D)** Plots depict the number of Aβ plaques marked with D54D2 and Amylo-Glo. The seeded Amylo-Glo-positive (≤50 µm) and growing cored plaques (≥200 µm) were measured. A plot depicts Lamp1-labeled dystrophic neurites as shown by the area ratio (Lamp1/D54D2) in each plaque in 5xFAD (sham) (10-month-old, n = 3, one male and two females) and Ift88-flox/flox; 5xFAD (10-month-old, n = 3, one male and two females), injected with AAV-pCD68-Cre. **(E)** Representative images of Aβ plaque (Amylo-Glo, blue)-associated microglia (Iba1, red) in the LS of 5xFAD (sham) (10-month-old, n = 3, one male and two females) and Ift88-flox/flox; 5xFAD mice (10-month-old, n = 3, one male and two females), injected with AAV-pCD68-Cre. **(F)** A plot depicts the number of microglia adjacent to each Aβ plaque. **(G)** Representative images of PSVue-550, CD63, and CD81 show the vesicular coverage in the Amylo-Glo-positive plaques of the cortex and the LS. **(H)** Plots depict the intensity of PSVue-550 that binds phosphatidylserine of EVs and EV markers, such as CD63 and CD81, in the cortex and the LS of 5xFAD (sham) (10-month-old, *n* = 3) and Ift88-flox/flox; 5xFAD (10-month-old, *n* = 3) injected with AAV-pCD68-Cre. **(D, F, H)** The data represent means ± SEM, and *p*-values were calculated by an unpaired two-tailed *t*-test. ****p < 0.0001, *p < 0.05. Scale bars = 100 ㎛ **(B)**; 10 ㎛ **(C, E, G)**.

To further investigate the role of microglial primary cilia in the development of AD, we utilized 5xFAD mice carrying the conditional Ift88-flox allele (5xFAD; Ift88-flox/flox). The chemokine receptor Cx3cr1 serves as a selective marker for microglia in the central nervous system. To examine the effects of endogenous Ift88 loss in microglia, we generated Cx3cr1-cre; Ift88-flox/flox; 5xFAD mice by crossing Cx3cr1-Cre mice (Yona et al., 2013) with 5xFAD; Ift88-flox/flox mice (Supplementary Figure S5A). We investigated the characteristics of Aβ plaques and dystrophic neurites in the LS of 3-month-old mice when the Aβ plaques started to occupy the region. The number of small-sized Amylo-Glo plaques (<50 µm) significantly increased, and there was a noticeable trend of increased Aβ plaques marked with D54D2 (Supplementary Figure S5B). Furthermore, the fluorescence intensity of GFAP and Iba1 was significantly elevated (Supplementary Figure S5C, D), indicating elevated gliosis possibly from the promoted EV secretion containing altered EV proteins from Ift88-inhibited microglia. To avoid the possible non-specific inhibition of Ift88 in the Cre-driver mouse line (Zhao et al., 2019), we also employed an intracranial injection of adeno-associated virus (AAV) carrying Cre recombinase under the control of the CD68 promoter (AAV-pCD68-Cre), which is active in activated microglial cells (Bodea et al., 2014). Figure 8A illustrates an experimental outline to investigate the function of microglial Ift88 in the axon terminal region of the vDG-LS tract and its impact on the pathogenesis of AD. We induced the loss of Ift88 in 5xFAD mice at 9 months of age through stereotaxic injection of AAV-pCD68-Cre. Immunohistochemical analysis of the LS in 5xFAD; Ift88-flox/flox mice injected with AAV-pCD68-Cre revealed pronounced Lamp1 signals, indicative of dystrophic neuronal membranes including axons, compared to 5xFAD mice injected with a sham control (Figures 8B,C). Notably, the LS of 5xFAD; Ift88-flox/flox mice injected with AAV-pCD68-Cre exhibited a significant increase in Aβ plaques (D54D2) and Amylo-Glo stained plaques, specifically in the small (<50 µm) and large (>200 µm) size ranges (Figures 8C,D). Considering that microglia lacking Ift88 tended to engulf and secrete more EVs containing Aβ, we hypothesized that the enlarged plaques may be contributed by microglial secretion, which also induced neuritic dystrophy adjacent to the plaques. Indeed, staining for Lamp1-positive dystrophic neurites adjacent to Aβ plaques revealed a significant increase in dystrophic neurites in 5xFAD mice with conditionally inhibited Ift88 expression in microglial cells (Figure 8D). These findings suggest that the loss of Ift88 in microglia may contribute to the increased occurrence of diffused and fibrillar Aβ plaques as well as dystrophic neurons affecting the axon projection. The toxic substances may also affect the dystrophy and recruitment of microglia to the Aβ plaques. Quantifying microglia near each plaque enabled us to examine the recognition and interaction of microglia with plaques in both 5xFAD and 5xFAD mice with Ift88-deficient microglia. We observed a reduced number of microglia located within 5 µm of the plaque in 5xFAD; Ift88-flox/flox mice injected with AAV-CD68-Cre compared to 5xFAD mice (Figures 8E,F). This finding suggests that the loss of microglial Ift88 affects the sensing and recruitment of microglia towards Aβ plaques. The facilitated EV secretion in Ift88-deficient microglia could aggravate the amyloidopathy via dampening of microglial primary cilia function and altering the extracellular proteostasis.

To investigate the dysregulated accumulation of EVs on the Aβ plaques in the cortex and the septum, we conducted immunohistochemistry analyses using 5xFAD (sham) and 5xFAD; Ift88-flox/flox mice injected with AAV-pCD68-Cre, employing anti-CD63 and anti-CD81, as well as the vesicle-specific dye PSVue-550. Interestingly, the intensities of CD63, CD81, and PSVue-550 were significantly elevated within the Aβ plaques in the LS of 5xFAD; Ift88-flox/flox mice injected with AAV-pCD68-Cre relatively to 5xFAD (sham) (Figures 8G,H). The markers of EVs such as CD63 and CD81 were also elevated in the cortical Aβ plaques after the inhibition of Ift88 with slight decreased signals of PSVue. Even though cortical microglia may behave differently than septal microglia, it is reasonable to assume that the lipid composition of the cortical plaques may confer resistance to PSVue staining, given that the EV markers were elevated in Aβ plaques in the LS, just as in the cortical plaques. These results provide compelling evidence supporting our initial idea that the absence of Ift88 in microglia may potentiate the secretion of EVs, potentially contributing to increased plaque formation.

## Discussion

AD risk genes such as TREM2, ABCA7, CD33, MS4A6A, CR1, INPP5D, *BIN1, PICALM, EPHA1, CD2AP, SORL1,* and *RIN3* are tightly linked to Aβ clearance via the endo-lysosomal pathway (Hansen et al., 2018; Lambert et al., 2022). EVs have been studied primarily to discover biomarkers of AD progression (Watson et al., 2019; Eren et al., 2020). In this study, we demonstrated that microglial primary cilia are involved in EV secretion, and that disruption of this process has a substantial effect on Aβ plaques and dystrophic neurites, highlighting the possibility that EV-related genes could be AD risk genes. Primary cilia could regulate the sensing of the recruitment of microglia toward the amyloidopathy, and impaired microglia aggravated the condition by altering the extracellular proteomic composition. It has been known that DAM-like microglia appear in the brain region at the time of postnatal cortical myelination (Hammond et al., 2019; Li et al., 2019). Our research also revealed the highly enriched presence of DAM-like microglia in the adult LS. The fornix, a bundle of hippocampal neuronal axons, targets the LS under the corpus callosum (Senova et al., 2020). These axons may contribute to the appearance of DAM-like microglia in the LS through the maintenance of axon structures and the removal of damaged myelin. Other fornix targets, such as the mammillary body, are also enriched for dystrophic neurites in the vicinity of Aβ plaques in 5xFAD mice (Canter et al., 2019). Future studies should determine whether the mammillary body-specific microglia are involved in amyloidopathy, which may strengthen the major contribution of the fornix and associated microglia to AD pathologies.

Maintenance of extracellular proteostasis, or a balanced proteome composition in the extracellular space, is essential for the proper functions of neurons and glial cells (Geraghty et al., 2021). In Ift88-deficient microglia, the secreted proteome was perturbed, especially increasing translation-related proteome in EVs, which may worsen the extracellular proteostasis and disrupt the compaction of Aβ plaques. Dystrophic neurites containing neuropathologic proteins such as Aβ aggregates and neurofibrillary tangles should be removed by nearby phagocytic cells to contain the spreading of damage (Gomez-Arboledas et al., 2018). DAM is localized close to dystrophic neurites containing APP and p-tau, and phagocytosis of the neurite may lead to extracellular Aβ plaque compaction following vesicular secretion of lysosomal contents. The compaction of Aβ plaques is protective for extracellular proteostasis by sequestrating toxic substances (Butler et al., 2021; Huang et al., 2021). The selective phagocytosis of extracellular amyloid proteins, dystrophic neurites, and EVs secreted by other cells by DAM is regulated by microglial membrane proteins and proteins controlling endo-lysosomal transport. It has been known that activated microglia highly express EV-specific tetraspanin proteins such as CD9, CD63, and CD81 (Keren-Shaul et al., 2017; Sousa et al., 2018). These proteins occupy specific membrane domains in EVs (Shoham et al., 2006; Umeda et al., 2020; Ma et al., 2021). Extracellular proteostasis could be affected by the altered proteomic composition of EVs as well as dysregulation of phagocytosis, endolysosomal degradation, and vesicular secretion. Thus, the biosynthesis of secretory vesicles and the selective loading of proteins for secretion are another signaling pathway of DAM that must be finely controlled. Recent single-cell analysis of microglia heterogeneity revealed that DAM subtypes are neuroprotective in a narrow window by maintaining their genomic signatures, which regulate containment of the damage (Keren-Shaul et al., 2017). In this study, we revealed that DAM are prone to generating vicious microglia, which are dysfunctional and excessively pro-inflammatory, by altering their secretome. Ift88 or primary cilia may regulate phagocytosis and EV secretion in microglia by sensing the extracellular milieu, such as the accumulation and spread of Aβ plaques in AD progression, which impose vulnerable conditions for the ciliated microglia. Even though we revealed Ift88 regulates EV secretion in microglial cells, the microglia in pathological conditions such as amyloidopathy and tauopathy also promote EVs with altered proteomic content, which should be addressed in future studies.

Lysosomal degradation of proteins and organelles is a highly conserved intracellular process for the removal of damaged components or the recycling of functional compartments. Intracellular accumulation of altered proteins interferes with normal cell functioning and has been linked to neurodegenerative diseases (Shen and Mizushima, 2014). Microglial lysosomal degradation is the key clearance pathway in the brain, however, overloading the microglial endo-lysosomal pathway leads to aggravation of neuropathology by failure of clearance and subsequent cellular death, leaving much more toxic debris (Gabande-Rodriguez et al., 2019). Endo-lysosomal components of microglia are vulnerable to aging and contribute to late-onset neurodegeneration (Van Acker et al., 2019). Thus, a clear understanding of the phagocytosis-exocytosis coupling in activated microglia is key to developing therapeutics for neurodegenerative diseases such as AD. Lysosome exocytosis carries molecules left over from degradation secretion out of cells. It is assumed that there are molecular switches balancing whether the lysosome recycles molecules without encapsulating secretory vesicles or *vice versa* (Andrews, 2000; Medina et al., 2011). The switch function is critical, especially in pathological conditions when microglial phagocytosis of extracellular debris increases intracellular demands responsible for breakdown in lysosomes. Incorrect clearance of phagocytosed debris and neuroinflammatory stimuli released from the secretory vesicles may jeopardize the condition, and repeated failure of the recycle itself may also interfere with the microglial viability. The prevention of microglial lysosomal burden is largely dependent on EV secretion and reuptake by healthy nearby phagocytic cells. For example, misfolded proteins released by dystrophic microglia should be reabsorbed by other glial cells in the vicinity. Without re-uptake and clearance, dystrophic microglia secrete toxic substances, which can cause the damage to spread. Microglial EVs are a major carrier of p-tau (Clayton et al., 2021; Zhu et al., 2022). The cellular mechanism underlying EV-mediated tau spreading may be a microglial defense involving the reduction of lysosomal burden via the secretion of EVs after ingesting p-tau-containing dystrophic neurites. The microglial regulation of lysosomal recycling or vesicular secretion could affect the microenvironment since microglial lysosomal failure may result in the accumulation of dead microglial cells with toxic substrates, which eventually worsens the burden of the clearance system. The coordination of microglial recycling and vesicle secretion is thus critical for the survival of the cell as well as tissue function. By ingesting and secreting toxic substances without complete clearance in the lysosome, the Ift88-deleted microglia may create a toxic environment. In this context, age-related dysfunction of microglial cilia may increase the AD risk associated with microglial EV secretion.

While chemokines such as ATP and CCL5 released from damaged neurites and glial processes are known to control microglial migration (Carbonell et al., 2005; Dou et al., 2012), intracellular signaling pathways regulating the directional migration are not clear. The axis of the primary cilia and the centrosome control cyclic AMP dynamics and PKA signaling in conjunction with AC3, which is enriched in the primary cilia, and regulate the migration of newly born neurons in the subventricular zone (Stoufflet et al., 2020). Recognition of the parenchymal milieu by microglia is mediated by cAMP in the filopodia of the microglial membrane (Bernier et al., 2019). Activation of intracellular adenylyl cyclase (AC) and PKA downstream of membrane receptors impaired microglial focal adhesion formation (Lee and Chung, 2009). The local cAMP dynamics regulated by the AC3, and centrosome may possibly regulate the migration of microglia toward the Aβ plaques. Dysregulated expansion of microglia damages the cells by limiting their clearance capacity in a small number of cells (Gomez-Nicola et al., 2013). Microglia deficient in Ift88 failed to cluster toward the Aβ plaques, resulting in the expansion of Aβ plaques and subsequent neurite dystrophies. The signaling pathways downstream of the primary cilia may control the microglial migration toward the Aβ plaque as a population, thereby allowing enough phagocytic cells to surround the extracellular toxic substrates. Overall, we provide evidence that microglia are heterogeneous in different brain regions and that microglial Ift88 may regulate phagocytic clearance and EV secretion in AD, possibly by sensing the extracellular milieu via the signaling of primary cilia. Targeting the microglial primary ciliary signaling system could therefore be a viable strategy for modulating neuroimmune responses in AD treatments.

## Data Availability

The proteomics data have been deposited with the ProteomeXchange Consortium via the PRIDE partner repository (identifier PXD043328) and single-cell RNA sequencing data can be accessible via the GEO repository (identifier GSE230240, GSE237776). The code and related raw data are available at https://github.com/choelab/Fronties-in-molecular-bioscience-2023.

## Supplementary material

SUPPLEMENTARY FIGURE S1 A heatmap illustrates the expression of marker genes used to annotate each cluster across down-sampled cells in columns and gene expression levels in rows.

SUPPLEMENTARY FIGURE S2 Subclusters of microglia representing the temporal and spatial heterogeneities **(A)** ∼ **(B)** UMAP plots depict the temporal **(A)** and spatial **(B)** distribution of microglial subsets. **(C)** A heatmap shows the expression of a group of marker genes differentially expressed in each microglial subclusters, representing down-sampled cells in columns and their gene expression in rows. **(D)** A dot plot depicts the expression of the canonical markers for microglia. The average expression is color-coded in a dot, whose size represents the percent of cells expressing the marker.

SUPPLEMENTARY FIGURE S3 scRNAseq characterization of all microglia acquired from five previously published datasets of GSE121654, GSE127893, GSE129788, GSE165306, and GSE166548 **(A)** UMAPs depict the projection of subclustered microglia from each published dataset **(B)** Color dots marked microglia derived from APP/PS1 and 5xFAD mice, including 75 microglia from 5xFAD mice and 584 microglia from APP/PS1 mice, compared to 119,274 microglia from WT mice (grey dots) **(C)** UMAP visualization of 36,013 microglial cells from clusters marked with homeostatic microglial genes or clusters overlapping with microglia from 5xFAD mice **(D)** A dot plot of 36,013 microglial cells with 17 clusters shows the expression of canonical microglial marker genes in each cluster.

SUPPLEMENTARY FIGURE S4 The volcano plots of the commonly upregulated proteins (adjust *p*-value <0.05, log2 (fold change) ≥ 0.5) or downregulated proteins (adjust *p*-value <0.05, log2 (fold change) ≤ −0.5) of CD81 binding proteins in cell lysates **(A)** and EVs **(B)** obtained from BV cells. These BV cells were transfected with siIft88 followed by treatment with Aβ (1 µM), in comparison to BV2 cells transfected with siCon and treated with Aβ (1 µM). In the volcano plot, representative proteins associated with each ontology term (Figure 7F) were marked with corresponding colors.

SUPPLEMENTARY FIGURE S5 Increased Aβ plaques and gliosis in the LS of Cx3cr1-Cre; Ift88-flox/flox; 5xFAD compared to the littermate 5xFAD mice at 3 months of age **(A)** Representative images of Aβ plaques (D54D2, white), plaque cores (Amylo-Glo, blue), and dystrophic neurites (Lamp1, red) in the LS of 5xFAD mice (two male and one female) and Cx3cr1-cre; Ift88-flox/flox; 5xFAD mice (three female) at 3 months old. **(B)** Plots depict the number of Aβ plaques marked by D54D2, Aβ plaques without Amylo-Glo-labeled cores, and small Aβ plaques-labeled with Amylo-Glo (≤50 µm) in the LS of 5xFAD mice (two male and one female) and Cx3cr1-cre; Ift88-flox/flox; 5xFAD mice (three female) at 3 months old. **(C)** Representative images of Aβ plaques (D54D2, white), plaque cores (Amylo-Glo, blue), astrocyte (GFAP, red), and microglia (Iba1, red) in the LS of 5xFAD mice (two male and one female) and Cx3cr1-cre; Ift88-flox/flox; 5xFAD mice (three female) at 3 months old. **(D)** Plots depict the increased gliosis presented with the increased intensity of GFAP and Iba1 in the LS of 5xFAD mice (two male and one female) and Cx3cr1-cre; Ift88-flox/flox; 5xFAD mice (three female) at 3 months old. **(B, D)** Data represent means ± SEM, and p-values were calculated by an unpaired two-tailed *t*-test (n = 4). ****p < 0.0001, **p < 0.01, *p < 0.05. Scale bars = 100 µm (A top, **(C)**; 10 µm (A middle and bottom).

SUPPLEMENTARY FILE S1 Differentially expressed genes on the MG-01 and the MG-03 clusters in comparison to the MG-02 in WT mice used in Figure 3D. Enriched pathway lists based on GO-CC, GO-BP, and Reactome used in Figure 3E using clusterProfiler.

SUPPLEMENTARY FILE S2 Differentially expressed proteins identified by mass spectrometry-based proteomic analysis of cell lysates and EVs from BV-2 microglial cells used in Figure 6C,D. Enriched pathway lists with Reactome, KEGG, GO-BP, and GO-CC used in Figure 6E,F using Enrichr.

SUPPLEMENTARY FILE S3 Differentially expressed proteins identified by mass spectrometry-based proteomic analysis of CD63-based immunoprecipitation of cell lysates and EVs from BV-2 microglial cells used in Figures 7C,D. Enriched pathway lists with Reactome, KEGG, GO-BP, and GO-CC used in Figure 7E using Enrichr.

SUPPLEMENTARY FILE S4 Differentially expressed proteins identified by mass spectrometry-based proteomic analysis of CD81-based immunoprecipitation of cell lysates and EVs from BV-2 microglial cells used in Supplementary Figure S4. Enriched pathway lists with Reactome, KEGG, GO-BP, and GO-CC used in Figure 7F using Enrichr.

## Abbreviations

AAV, adeno-associated virus; AC3, adenylate cyclase type III; AD, Alzheimer’s disease; ATM, axon tract microglia; Aβ, amyloid-beta; Co-IP, Co-immunoprecipitation; CP, caudate putamen; Cx3cr1, CX3 chemokine receptor 1; DAM, disease-associated microglia; EVs, extracellular vesicles; GSEA, gene-set enrichment analysis; Hb, Habenula; Hif-1a, hypoxia-inducible factor-1α; Ift88, intraflagellar transport particle 88; IRM, interferon-responsive microglia; LCMS, liquid chromatography-mass spectrometry; LS, lateral septum; Lyz2, Lysozyme M; mPFC, medial prefrontal cortex; NAc, nucleus accumbens; NTA, nanoparticle tracking analysis; P2ry12, P2Y purinoreceptor 12; pHrodo-Aβ, Ph-sensitive dye-conjugated aβ; Tmem119, transmembrane protein 119; UMAP, uniform manifold approximation and projection; vDG, ventral dentate gyrus; WT, wild-type; GO, gene ontology; PPI, protein-protein interaction.

## References

[B1] Aghaizu N. D. Jolly S. Samra S. K. Kalmar B. Craessaerts K. Greensmith L. (2023). Microglial Expression of the Wnt Signaling Modulator DKK2 Differs between Human Alzheimer's Disease Brains and Mouse Neurodegeneration Models. eNeuro 10 (1), ENEURO.0306–22.2022. 10.1523/ENEURO.0306-22.2022 36599670 PMC9836029

[B2] Alquicira-Hernandez J. Powell J. E. (2021). Nebulosa recovers single-cell gene expression signals by kernel density estimation. Bioinformatics 37 (16), 2485–2487. 10.1093/bioinformatics/btab003 33459785

[B3] Andreatta M. Carmona S. J. (2021). UCell: robust and scalable single-cell gene signature scoring. Comput. Struct. Biotechnol. J. 19, 3796–3798. 10.1016/j.csbj.2021.06.043 34285779 PMC8271111

[B4] Andrews N. W. (2000). Regulated secretion of conventional lysosomes. Trends Cell Biol. 10 (8), 316–321. 10.1016/s0962-8924(00)01794-3 10884683

[B5] Barthet G. Mulle C. (2020). Presynaptic failure in Alzheimer's disease. Prog. Neurobiol. 194, 101801. 10.1016/j.pneurobio.2020.101801 32428558

[B6] Berbari N. F. Lewis J. S. Bishop G. A. Askwith C. C. Mykytyn K. (2008). Bardet-Biedl syndrome proteins are required for the localization of G protein-coupled receptors to primary cilia. Proc. Natl. Acad. Sci. U. S. A. 105 (11), 4242–4246. 10.1073/pnas.0711027105 18334641 PMC2393805

[B7] Berditchevski F. Odintsova E. (2007). Tetraspanins as regulators of protein trafficking. Traffic 8 (2), 89–96. 10.1111/j.1600-0854.2006.00515.x 17181773

[B8] Bernier L. P. Bohlen C. J. York E. M. Choi H. B. Kamyabi A. Dissing-Olesen L. (2019). Nanoscale Surveillance of the Brain by Microglia via cAMP-Regulated Filopodia. Cell Rep. 27 (10), 2895–2908. 10.1016/j.celrep.2019.05.010 31167136

[B9] Bishop G. A. Berbari N. F. Lewis J. Mykytyn K. (2007). Type III adenylyl cyclase localizes to primary cilia throughout the adult mouse brain. J. Comp. Neurol. 505 (5), 562–571. 10.1002/cne.21510 17924533

[B10] Bodea L. G. Wang Y. Linnartz-Gerlach B. Kopatz J. Sinkkonen L. Musgrove R. (2014). Neurodegeneration by activation of the microglial complement-phagosome pathway. J. Neurosci. 34 (25), 8546–8556. 10.1523/JNEUROSCI.5002-13.2014 24948809 PMC6608212

[B11] Butler A. Hoffman P. Smibert P. Papalexi E. Satija R. (2018). Integrating single-cell transcriptomic data across different conditions, technologies, and species. Nat. Biotechnol. 36 (5), 411–420. 10.1038/nbt.4096 29608179 PMC6700744

[B12] Butler C. A. Popescu A. S. Kitchener E. J. A. Allendorf D. H. Puigdellivol M. Brown G. C. (2021). Microglial phagocytosis of neurons in neurodegeneration, and its regulation. J. Neurochem. 158 (3), 621–639. 10.1111/jnc.15327 33608912

[B13] Butovsky O. Jedrychowski M. P. Moore C. S. Cialic R. Lanser A. J. Gabriely G. (2014). Identification of a unique TGF-beta-dependent molecular and functional signature in microglia. Nat. Neurosci. 17 (1), 131–143. 10.1038/nn.3599 24316888 PMC4066672

[B14] Canter R. G. Huang W. C. Choi H. Wang J. Watson L. A. Yao C. G. (2019). 3D mapping reveals network-specific amyloid progression and subcortical susceptibility in mice. Commun. Biol. 2, 360. 10.1038/s42003-019-0599-8 31602409 PMC6778135

[B15] Cao J. Spielmann M. Qiu X. Huang X. Ibrahim D. M. Hill A. J. (2019). The single-cell transcriptional landscape of mammalian organogenesis. Nature 566 (7745), 496–502. 10.1038/s41586-019-0969-x 30787437 PMC6434952

[B16] Carbonell W. S. Murase S. Horwitz A. F. Mandell J. W. (2005). Migration of perilesional microglia after focal brain injury and modulation by CC chemokine receptor 5: an *in situ* time-lapse confocal imaging study. J. Neurosci. 25 (30), 7040–7047. 10.1523/JNEUROSCI.5171-04.2005 16049180 PMC6724831

[B17] Clayton K. Delpech J. C. Herron S. Iwahara N. Ericsson M. Saito T. (2021). Plaque associated microglia hyper-secrete extracellular vesicles and accelerate tau propagation in a humanized APP mouse model. Mol. Neurodegener. 16 (1), 18. 10.1186/s13024-021-00440-9 33752701 PMC7986521

[B18] Cohn W. Melnik M. Huang C. Teter B. Chandra S. Zhu C. (2021). Multi-Omics Analysis of Microglial Extracellular Vesicles From Human Alzheimer's Disease Brain Tissue Reveals Disease-Associated Signatures. Front. Pharmacol. 12, 766082. 10.3389/fphar.2021.766082 34925024 PMC8675946

[B19] Conduit S. E. Vanhaesebroeck B. (2020). Phosphoinositide lipids in primary cilia biology. Biochem. J. 477 (18), 3541–3565. 10.1042/Bcj20200277 32970140 PMC7518857

[B20] Damani M. R. Zhao L. Fontainhas A. M. Amaral J. Fariss R. N. Wong W. T. (2011). Age-related alterations in the dynamic behavior of microglia. Aging Cell 10 (2), 263–276. 10.1111/j.1474-9726.2010.00660.x 21108733 PMC3056927

[B21] Delous M. Baala L. Salomon R. Laclef C. Vierkotten J. Tory K. (2007). The ciliary gene RPGRIP1L is mutated in cerebello-oculo-renal syndrome (Joubert syndrome type B) and Meckel syndrome. Nat. Genet. 39 (7), 875–881. 10.1038/ng2039 17558409

[B22] Doncheva N. T. Morris J. H. Gorodkin J. Jensen L. J. (2019). Cytoscape StringApp: network Analysis and Visualization of Proteomics Data. J. Proteome Res. 18 (2), 623–632. 10.1021/acs.jproteome.8b00702 30450911 PMC6800166

[B23] Dou Y. Wu H. J. Li H. Q. Qin S. Wang Y. E. Li J. (2012). Microglial migration mediated by ATP-induced ATP release from lysosomes. Cell Res. 22 (6), 1022–1033. 10.1038/cr.2012.10 22231629 PMC3367529

[B24] Eitan E. Suire C. Zhang S. Mattson M. P. (2016). Impact of lysosome status on extracellular vesicle content and release. Ageing Res. Rev. 32, 65–74. 10.1016/j.arr.2016.05.001 27238186 PMC5154730

[B25] Eren E. Hunt J. F. V. Shardell M. Chawla S. Tran J. Gu J. (2020). Extracellular vesicle biomarkers of Alzheimer's disease associated with sub-clinical cognitive decline in late middle age. Alzheimers Dement. 16 (9), 1293–1304. 10.1002/alz.12130 32588967 PMC7984100

[B26] Finetti F. Cassioli C. Cianfanelli V. Onnis A. Paccagnini E. Kabanova A. (2020). The intraflagellar transport protein IFT20 controls lysosome biogenesis by regulating the post-Golgi transport of acid hydrolases. Cell Death Differ. 27 (1), 310–328. 10.1038/s41418-019-0357-y 31142807 PMC7205998

[B27] Finetti F. Cassioli C. Cianfanelli V. Zevolini F. Onnis A. Gesualdo M. (2021). The Intraflagellar Transport Protein IFT20 Recruits ATG16L1 to Early Endosomes to Promote Autophagosome Formation in T Cells. Front. Cell Dev. Biol. 9, 634003. 10.3389/fcell.2021.634003 33829015 PMC8019791

[B28] Finetti F. Patrussi L. Masi G. Onnis A. Galgano D. Lucherini O. M. (2014). Specific recycling receptors are targeted to the immune synapse by the intraflagellar transport system. J. Cell Sci. 127 (9), 1924–1937. 10.1242/jcs.139337 24554435 PMC4004972

[B29] Gabande-Rodriguez E. Perez-Canamas A. Soto-Huelin B. Mitroi D. N. Sanchez-Redondo S. Martinez-Saez E. (2019). Lipid-induced lysosomal damage after demyelination corrupts microglia protective function in lysosomal storage disorders. EMBO J. 38 (2), e99553. 10.15252/embj.201899553 30530526 PMC6331723

[B30] Gao C. H. Yu G. Cai P. (2021). ggVennDiagram: an Intuitive, Easy-to-Use, and Highly Customizable R Package to Generate Venn Diagram. Front. Genet. 12, 706907. 10.3389/fgene.2021.706907 34557218 PMC8452859

[B31] Geraghty N. J. Satapathy S. Kelly M. Cheng F. Lee A. Wilson M. R. (2021). Expanding the family of extracellular chaperones: identification of human plasma proteins with chaperone activity. Protein Sci. 30 (11), 2272–2286. 10.1002/pro.4189 34553437 PMC8521303

[B32] Germain P. L. Lun A. Garcia Meixide C. Macnair W. Robinson M. D. (2021). Doublet identification in single-cell sequencing data using scDblFinder. F1000Res 10, 979. 10.12688/f1000research.73600.2 35814628 PMC9204188

[B33] Gomez-Arboledas A. Davila J. C. Sanchez-Mejias E. Navarro V. Nunez-Diaz C. Sanchez-Varo R. (2018). Phagocytic clearance of presynaptic dystrophies by reactive astrocytes in Alzheimer's disease. Glia 66 (3), 637–653. 10.1002/glia.23270 29178139 PMC5814816

[B34] Gomez-Nicola D. Fransen N. L. Suzzi S. Perry V. H. (2013). Regulation of microglial proliferation during chronic neurodegeneration. J. Neurosci. 33 (6), 2481–2493. 10.1523/JNEUROSCI.4440-12.2013 23392676 PMC6619184

[B35] Gotzl J. K. Colombo A. V. Fellerer K. Reifschneider A. Werner G. Tahirovic S. (2018). Early lysosomal maturation deficits in microglia triggers enhanced lysosomal activity in other brain cells of progranulin knockout mice. Mol. Neurodegener. 13 (1), 48. 10.1186/s13024-018-0281-5 30180904 PMC6123925

[B36] Grubman A. Choo X. Y. Chew G. Ouyang J. F. Sun G. Croft N. P. (2021). Transcriptional signature in microglia associated with Aβ plaque phagocytosis. Nat. Commun. 12 (1), 3015. 10.1038/s41467-021-23111-1 34021136 PMC8140091

[B37] Guemez-Gamboa A. Coufal N. G. Gleeson J. G. (2014). Primary cilia in the developing and mature brain. Neuron 82 (3), 511–521. 10.1016/j.neuron.2014.04.024 24811376 PMC4104280

[B38] Hammond T. R. Dufort C. Dissing-Olesen L. Giera S. Young A. Wysoker A. (2019). Single-Cell RNA Sequencing of Microglia throughout the Mouse Lifespan and in the Injured Brain Reveals Complex Cell-State Changes. Immunity 50 (1), 253–271. 10.1016/j.immuni.2018.11.004 30471926 PMC6655561

[B39] Hansen D. V. Hanson J. E. Sheng M. (2018). Microglia in Alzheimer's disease. J. Cell Biol. 217 (2), 459–472. 10.1083/jcb.201709069 29196460 PMC5800817

[B40] Hao Y. Hao S. Andersen-Nissen E. Mauck W. M. 3rd Zheng S. Butler A. (2021). Integrated analysis of multimodal single-cell data. Cell 184 (13), 3573–3587.e29. 10.1016/j.cell.2021.04.048 34062119 PMC8238499

[B41] Hasenpusch-Theil K. Theil T. (2021). The Multifaceted Roles of Primary Cilia in the Development of the Cerebral Cortex. Front. Cell Dev. Biol. 9, 630161. 10.3389/fcell.2021.630161 33604340 PMC7884624

[B42] Hesketh S. J. Mukhopadhyay A. G. Nakamura D. Toropova K. Roberts A. J. (2022). IFT-A structure reveals carriages for membrane protein transport into cilia. Cell 185 (26), 4971–4985.e16. 10.1016/j.cell.2022.11.010 36462505

[B43] Hessvik N. P. Llorente A. (2018). Current knowledge on exosome biogenesis and release. Cell Mol. Life Sci. 75 (2), 193–208. 10.1007/s00018-017-2595-9 28733901 PMC5756260

[B44] Higuchi-Sanabria R. Shen K. Kelet N. Frankino P. A. Durieux J. Bar-Ziv R. (2020). Lysosomal recycling of amino acids affects ER quality control. Sci. Adv. 6 (26), eaaz9805. 10.1126/sciadv.aaz9805 32637599 PMC7319768

[B45] Hsiao Y. C. Tuz K. Ferland R. J. (2012). Trafficking in and to the primary cilium. Cilia 1 (1), 4. 10.1186/2046-2530-1-4 23351793 PMC3541539

[B46] Hu X. Y. Crick S. L. Bu G. J. Frieden C. Pappu R. V. Lee J. M. (2009). Amyloid seeds formed by cellular uptake, concentration, and aggregation of the amyloid-beta peptide. Proc. Natl. Acad. Sci. U. S. A. 106 (48), 20324–20329. 10.1073/pnas.0911281106 19910533 PMC2787156

[B47] Hu Y. B. Dammer E. B. Ren R. J. Wang G. (2015). The endosomal-lysosomal system: from acidification and cargo sorting to neurodegeneration. Transl. Neurodegener. 4, 18. 10.1186/s40035-015-0041-1 26448863 PMC4596472

[B48] Huang Y. Happonen K. E. Burrola P. G. O'Connor C. Hah N. Huang L. (2021). Microglia use TAM receptors to detect and engulf amyloid beta plaques. Nat. Immunol. 22 (5), 586–594. 10.1038/s41590-021-00913-5 33859405 PMC8102389

[B49] Jang J. Yeo S. Baek S. Jung H. J. Lee M. S. Choi S. H. (2023). Abnormal accumulation of extracellular vesicles in hippocampal dystrophic axons and regulation by the primary cilia in Alzheimer's disease. Acta Neuropathol. Commun. 11 (1), 142. 10.1186/s40478-023-01637-3 37667395 PMC10478284

[B50] Keren-Shaul H. Spinrad A. Weiner A. Matcovitch-Natan O. Dvir-Szternfeld R. Ulland T. K. (2017). A Unique Microglia Type Associated with Restricting Development of Alzheimer's Disease. Cell 169 (7), 1276–1290. 10.1016/j.cell.2017.05.018 28602351

[B51] Kim H. Xu H. X. Yao Q. Li W. Z. Huang Q. Outeda P. (2014). Ciliary membrane proteins traffic through the Golgi via a Rabep1/GGA1/Arl3-dependent mechanism. Nat. Commun. 5, ARTN 5482. 10.1038/ncomms6482 PMC4237283 25405894

[B52] Klein A. M. Mazutis L. Akartuna I. Tallapragada N. Veres A. Li V. (2015). Droplet barcoding for single-cell transcriptomics applied to embryonic stem cells. Cell 161 (5), 1187–1201. 10.1016/j.cell.2015.04.044 26000487 PMC4441768

[B53] Korsunsky I. Millard N. Fan J. Slowikowski K. Zhang F. Wei K. (2019). Fast, sensitive and accurate integration of single-cell data with Harmony. Nat. Methods 16 (12), 1289–1296. 10.1038/s41592-019-0619-0 31740819 PMC6884693

[B54] Krasemann S. Madore C. Cialic R. Baufeld C. Calcagno N. El Fatimy R. (2017). The TREM2-APOE Pathway Drives the Transcriptional Phenotype of Dysfunctional Microglia in Neurodegenerative Diseases. Immunity 47 (3), 566–581. 10.1016/j.immuni.2017.08.008 28930663 PMC5719893

[B55] Krause G. J. Diaz A. Jafari M. Khawaja R. R. Agullo-Pascual E. Santiago-Fernandez O. (2022). Reduced endosomal microautophagy activity in aging associates with enhanced exocyst-mediated protein secretion. Aging Cell 21 (10), e13713. 10.1111/acel.13713 36116133 PMC9577956

[B56] Lall D. Lorenzini I. Mota T. A. Bell S. Mahan T. E. Ulrich J. D. (2021). C9orf72 deficiency promotes microglial-mediated synaptic loss in aging and amyloid accumulation. Neuron 109 (14), 2275–2291.e8. 10.1016/j.neuron.2021.05.020 34133945 PMC8298293

[B57] Lambert E. Saha O. Landeira B. S. de Farias A. R. M. Hermant X. Carrier A. (2022). The Alzheimer susceptibility gene BIN1 induces isoform-dependent neurotoxicity through early endosome defects. Acta Neuropathol. Commun. 10 (1), ARTN 4. 10.1186/s40478-021-01285-5 PMC8742943 34998435

[B58] Lee S. Chung C. Y. (2009). Role of VASP phosphorylation for the regulation of microglia chemotaxis via the regulation of focal adhesion formation/maturation. Mol. Cell Neurosci. 42 (4), 382–390. 10.1016/j.mcn.2009.08.010 19733667 PMC3904500

[B59] Lewis K. B. S. R. E. T. B. O. A. G. M. (2023). EnhancedVolcano: publication-ready volcano plots with enhanced colouring and labeling". R package version 1.18.0. Release: Bioconductor version. (3.17.

[B60] Li Q. Cheng Z. Zhou L. Darmanis S. Neff N. F. Okamoto J. (2019). Developmental Heterogeneity of Microglia and Brain Myeloid Cells Revealed by Deep Single-Cell RNA Sequencing. Neuron 101 (2), 207–223. 10.1016/j.neuron.2018.12.006 30606613 PMC6336504

[B61] Ma S. Mangala L. S. Hu W. Bayaktar E. Yokoi A. Hu W. (2021). CD63-mediated cloaking of VEGF in small extracellular vesicles contributes to anti-VEGF therapy resistance. Cell Rep. 36 (7), 109549. 10.1016/j.celrep.2021.109549 34407412 PMC8422976

[B62] Martin E. Boucher C. Fontaine B. Delarasse C. (2017). Distinct inflammatory phenotypes of microglia and monocyte-derived macrophages in Alzheimer's disease models: effects of aging and amyloid pathology. Aging Cell 16 (1), 27–38. 10.1111/acel.12522 27723233 PMC5242297

[B63] Medina D. L. Fraldi A. Bouche V. Annunziata F. Mansueto G. Spampanato C. (2011). Transcriptional activation of lysosomal exocytosis promotes cellular clearance. Dev. Cell 21 (3), 421–430. 10.1016/j.devcel.2011.07.016 21889421 PMC3173716

[B64] Mohieldin A. M. Pala R. Beuttler R. Moresco J. J. Yates J. R. 3rd Nauli S. M. (2021). Ciliary extracellular vesicles are distinct from the cytosolic extracellular vesicles. J. Extracell. Vesicles 10 (6), e12086. 10.1002/jev2.12086 33936569 PMC8077156

[B65] Muraoka S. DeLeo A. M. Sethi M. K. Yukawa-Takamatsu K. Yang Z. Ko J. (2020). Proteomic and biological profiling of extracellular vesicles from Alzheimer's disease human brain tissues. Alzheimers Dement. 16 (6), 896–907. 10.1002/alz.12089 32301581 PMC7293582

[B66] Muraoka S. Jedrychowski M. P. Iwahara N. Abdullah M. Onos K. D. Keezer K. J. (2021). Enrichment of Neurodegenerative Microglia Signature in Brain-Derived Extracellular Vesicles Isolated from Alzheimer's Disease Mouse Models. J. Proteome Res. 20 (3), 1733–1743. 10.1021/acs.jproteome.0c00934 33534581 PMC7944570

[B67] Novas R. Cardenas-Rodriguez M. Lepanto P. Fabregat M. Rodao M. Fariello M. I. (2018). Kinesin 1 regulates cilia length through an interaction with the Bardet-Biedl syndrome related protein CCDC28B. Sci. Rep. 8 (1), 3019. 10.1038/s41598-018-21329-6 29445114 PMC5813027

[B68] Onnis A. Finetti F. Baldari C. T. (2016). Vesicular Trafficking to the Immune Synapse: how to Assemble Receptor-Tailored Pathways from a Basic Building Set. Front. Immunol. 7, 50. 10.3389/fimmu.2016.00050 26913036 PMC4753310

[B69] Pampliega O. Cuervo A. M. (2016). Autophagy and primary cilia: dual interplay. Curr. Opin. Cell Biol. 39, 1–7. 10.1016/j.ceb.2016.01.008 26826446 PMC4733852

[B70] Parenti G. Medina D. L. Ballabio A. (2021). The rapidly evolving view of lysosomal storage diseases. EMBO Mol. Med. 13 (2), e12836. 10.15252/emmm.202012836 33459519 PMC7863408

[B71] Pedersen L. B. Mogensen J. B. Christensen S. T. (2016). Endocytic Control of Cellular Signaling at the Primary Cilium. Trends Biochem. Sci. 41 (9), 784–797. 10.1016/j.tibs.2016.06.002 27364476

[B72] Pike C. J. Burdick D. Walencewicz A. J. Glabe C. G. Cotman C. W. (1993). Neurodegeneration induced by beta-amyloid peptides *in vitro*: the role of peptide assembly state. J. Neurosci. 13 (4), 1676–1687. 10.1523/JNEUROSCI.13-04-01676.1993 8463843 PMC6576726

[B73] Ribeiro Xavier A. L. Kress B. T. Goldman S. A. Lacerda de Menezes J. R. Nedergaard M. (2015). A Distinct Population of Microglia Supports Adult Neurogenesis in the Subventricular Zone. J. Neurosci. 35 (34), 11848–11861. 10.1523/JNEUROSCI.1217-15.2015 26311768 PMC4549398

[B74] Safaiyan S. Besson-Girard S. Kaya T. Cantuti-Castelvetri L. Liu L. Ji H. (2021). White matter aging drives microglial diversity. Neuron 109 (7), 1100–1117.e10. 10.1016/j.neuron.2021.01.027 33606969

[B75] Sala Frigerio C. Wolfs L. Fattorelli N. Thrupp N. Voytyuk I. Schmidt I. (2019). The Major Risk Factors for Alzheimer's Disease: age, Sex, and Genes Modulate the Microglia Response to Aβ Plaques. Cell Rep. 27 (4), 1293–1306. 10.1016/j.celrep.2019.03.099 31018141 PMC7340153

[B76] Satoh J. Kino Y. Asahina N. Takitani M. Miyoshi J. Ishida T. (2016). TMEM119 marks a subset of microglia in the human brain. Neuropathology 36 (1), 39–49. 10.1111/neup.12235 26250788

[B77] Schafer D. P. Stevens B. (2015). Microglia Function in Central Nervous System Development and Plasticity. Cold Spring Harb. Perspect. Biol. 7 (10), a020545. 10.1101/cshperspect.a020545 26187728 PMC4588063

[B78] Selkoe D. J. (2002). Alzheimer's disease is a synaptic failure. Science 298 (5594), 789–791. 10.1126/science.1074069 12399581

[B79] Senova S. Fomenko A. Gondard E. Lozano A. M. (2020). Anatomy and function of the fornix in the context of its potential as a therapeutic target. J. Neurol. Neurosurg. Psychiatry 91 (5), 547–559. 10.1136/jnnp-2019-322375 32132227 PMC7231447

[B80] Settembre C. Zoncu R. Medina D. L. Vetrini F. Erdin S. Erdin S. (2012). A lysosome-to-nucleus signalling mechanism senses and regulates the lysosome via mTOR and TFEB. EMBO J. 31 (5), 1095–1108. 10.1038/emboj.2012.32 22343943 PMC3298007

[B81] Shannon P. Markiel A. Ozier O. Baliga N. S. Wang J. T. Ramage D. (2003). Cytoscape: a software environment for integrated models of biomolecular interaction networks. Genome Res. 13 (11), 2498–2504. 10.1101/gr.1239303 14597658 PMC403769

[B82] Shen H. M. Mizushima N. (2014). At the end of the autophagic road: an emerging understanding of lysosomal functions in autophagy. Trends Biochem. Sci. 39 (2), 61–71. 10.1016/j.tibs.2013.12.001 24369758

[B83] Shoham T. Rajapaksa R. Kuo C. C. Haimovich J. Levy S. (2006). Building of the tetraspanin web: distinct structural domains of CD81 function in different cellular compartments. Mol. Cell Biol. 26 (4), 1373–1385. 10.1128/MCB.26.4.1373-1385.2006 16449649 PMC1367195

[B84] Sousa C. Golebiewska A. Poovathingal S. K. Kaoma T. Pires-Afonso Y. Martina S. (2018). Single-cell transcriptomics reveals distinct inflammation-induced microglia signatures. EMBO Rep. 19 (11), e46171. 10.15252/embr.201846171 30206190 PMC6216255

[B85] Stinchcombe J. C. Randzavola L. O. Angus K. L. Mantell J. M. Verkade P. Griffiths G. M. (2015). Mother Centriole Distal Appendages Mediate Centrosome Docking at the Immunological Synapse and Reveal Mechanistic Parallels with Ciliogenesis. Curr. Biol. 25 (24), 3239–3244. 10.1016/j.cub.2015.10.028 26670998 PMC4691242

[B86] Stoufflet J. Chaulet M. Doulazmi M. Fouquet C. Dubacq C. Metin C. (2020). Primary cilium-dependent cAMP/PKA signaling at the centrosome regulates neuronal migration. Sci. Adv. 6 (36), ARTN eaba3992. 10.1126/sciadv.aba3992 PMC7467704 32917588

[B87] Stuart T. Butler A. Hoffman P. Hafemeister C. Papalexi E. Mauck W. M. 3rd (2019). Comprehensive Integration of Single-Cell Data. Cell 177 (7), 1888–1902. 10.1016/j.cell.2019.05.031 31178118 PMC6687398

[B88] Tao B. Bu S. Yang Z. Siroky B. Kappes J. C. Kispert A. (2009). Cystin localizes to primary cilia via membrane microdomains and a targeting motif. J. Am. Soc. Nephrol. 20 (12), 2570–2580. 10.1681/ASN.2009020188 19850956 PMC2794227

[B89] Teng F. Fussenegger M. (2020). Shedding Light on Extracellular Vesicle Biogenesis and Bioengineering. Adv. Sci. (Weinh) 8 (1), 2003505. 10.1002/advs.202003505 33437589 PMC7788585

[B90] Umeda R. Satouh Y. Takemoto M. Nakada-Nakura Y. Liu K. Yokoyama T. (2020). Structural insights into tetraspanin CD9 function. Nat. Commun. 11 (1), 1606. 10.1038/s41467-020-15459-7 32231207 PMC7105497

[B91] Van Acker Z. P. Bretou M. Annaert W. (2019). Endo-lysosomal dysregulations and late-onset Alzheimer's disease: impact of genetic risk factors. Mol. Neurodegener. 14 (1), 20. 10.1186/s13024-019-0323-7 31159836 PMC6547588

[B92] van Dam T. J. P. Kennedy J. van der Lee R. de Vrieze E. Wunderlich K. A. Rix S. (2019). CiliaCarta: an integrated and validated compendium of ciliary genes. PLoS One 14 (5), e0216705. 10.1371/journal.pone.0216705 31095607 PMC6522010

[B93] Van den Berge K. Roux de Bezieux H. Street K. Saelens W. Cannoodt R. Saeys Y. (2020). Trajectory-based differential expression analysis for single-cell sequencing data. Nat. Commun. 11 (1), 1201. 10.1038/s41467-020-14766-3 32139671 PMC7058077

[B94] Vivar O. I. Masi G. Carpier J. M. Magalhaes J. G. Galgano D. Pazour G. J. (2016). IFT20 controls LAT recruitment to the immune synapse and T-cell activation *in vivo* . Proc. Natl. Acad. Sci. U. S. A. 113 (2), 386–391. 10.1073/pnas.1513601113 26715756 PMC4720349

[B95] Watson L. S. Hamlett E. D. Stone T. D. Sims-Robinson C. (2019). Neuronally derived extracellular vesicles: an emerging tool for understanding Alzheimer's disease. Mol. Neurodegener. 14 (1), 22. 10.1186/s13024-019-0317-5 31182115 PMC6558712

[B96] Wheway G. Nazlamova L. Hancock J. T. (2018). Signaling through the Primary Cilium. Front. Cell Dev. Biol. 6, 8. 10.3389/fcell.2018.00008 29473038 PMC5809511

[B97] Wickham H. (2016). “ggplot2: elegant Graphics for Data Analysis,” in Use R!. 2nd ed. (Cham: Springer International Publishing : Imprint: Springer).

[B98] Wu T. Hu E. Xu S. Chen M. Guo P. Dai Z. (2021). clusterProfiler 4.0: A universal enrichment tool for interpreting omics data. Innov. (Camb) 2 (3), 100141. 10.1016/j.xinn.2021.100141 PMC8454663 34557778

[B99] Ximerakis M. Lipnick S. L. Innes B. T. Simmons S. K. Adiconis X. Dionne D. (2019). Single-cell transcriptomic profiling of the aging mouse brain. Nat. Neurosci. 22 (10), 1696–1708. 10.1038/s41593-019-0491-3 31551601

[B100] Yajima A. M. G. F. M. (2023). Mast: model-Based analysis of single cell transcriptomics". R package version 1.26.0. Release: Bioconductor version. (3.17.

[B101] Ye X. Zhu M. Che X. Wang H. Liang X. J. Wu C. (2020). Lipopolysaccharide induces neuroinflammation in microglia by activating the MTOR pathway and downregulating Vps34 to inhibit autophagosome formation. J. Neuroinflammation 17 (1), 18. 10.1186/s12974-019-1644-8 31926553 PMC6954631

[B102] Yona S. Kim K. W. Wolf Y. Mildner A. Varol D. Breker M. (2013). Fate mapping reveals origins and dynamics of monocytes and tissue macrophages under homeostasis. Immunity 38 (1), 79–91. 10.1016/j.immuni.2012.12.001 23273845 PMC3908543

[B103] Yu G. Wang L. G. Han Y. He Q. Y. (2012). clusterProfiler: an R package for comparing biological themes among gene clusters. OMICS 16 (5), 284–287. 10.1089/omi.2011.0118 22455463 PMC3339379

[B104] Zhang X. Smits A. H. van Tilburg G. B. Ovaa H. Huber W. Vermeulen M. (2018). Proteome-wide identification of ubiquitin interactions using UbIA-MS. Nat. Protoc. 13 (3), 530–550. 10.1038/nprot.2017.147 29446774

[B105] Zhao X. F. Alam M. M. Liao Y. Huang T. Mathur R. Zhu X. (2019). Targeting Microglia Using Cx3cr1-Cre Lines: revisiting the Specificity. eNeuro 6 (4), ENEURO.0114–19.2019. 10.1523/ENEURO.0114-19.2019 31201215 PMC6620394

[B106] Zhu B. Liu Y. Hwang S. Archuleta K. Huang H. J. Campos A. (2022). Trem2 deletion enhances tau dispersion and pathology through microglia exosomes. Mol. Neurodegener. 17 (1), ARTN 58. 10.1186/s13024-022-00562-8 PMC9438095 36056435

[B107] Zilionis R. Nainys J. Veres A. Savova V. Zemmour D. Klein A. M. (2017). Single-cell barcoding and sequencing using droplet microfluidics. Nat. Protoc. 12 (1), 44–73. 10.1038/nprot.2016.154 27929523

[B108] Zuo X. Kwon S. H. Janech M. G. Dang Y. Lauzon S. D. Fogelgren B. (2019). Primary cilia and the exocyst are linked to urinary extracellular vesicle production and content. J. Biol. Chem. 294 (50), 19099–19110. 10.1074/jbc.RA119.009297 31694916 PMC6916495

