# Supplementary figures and images for: Primary cilia-mediated regulation of microglial secretion in Alzheimer’s disease
Source: Front Mol Biosci. 2023 Oct 23;10:1250335. doi: 10.3389/fmolb.2023.1250335 (PMC10627801; doi:10.3389/fmolb.2023.1250335)

Supp. Fig. 1

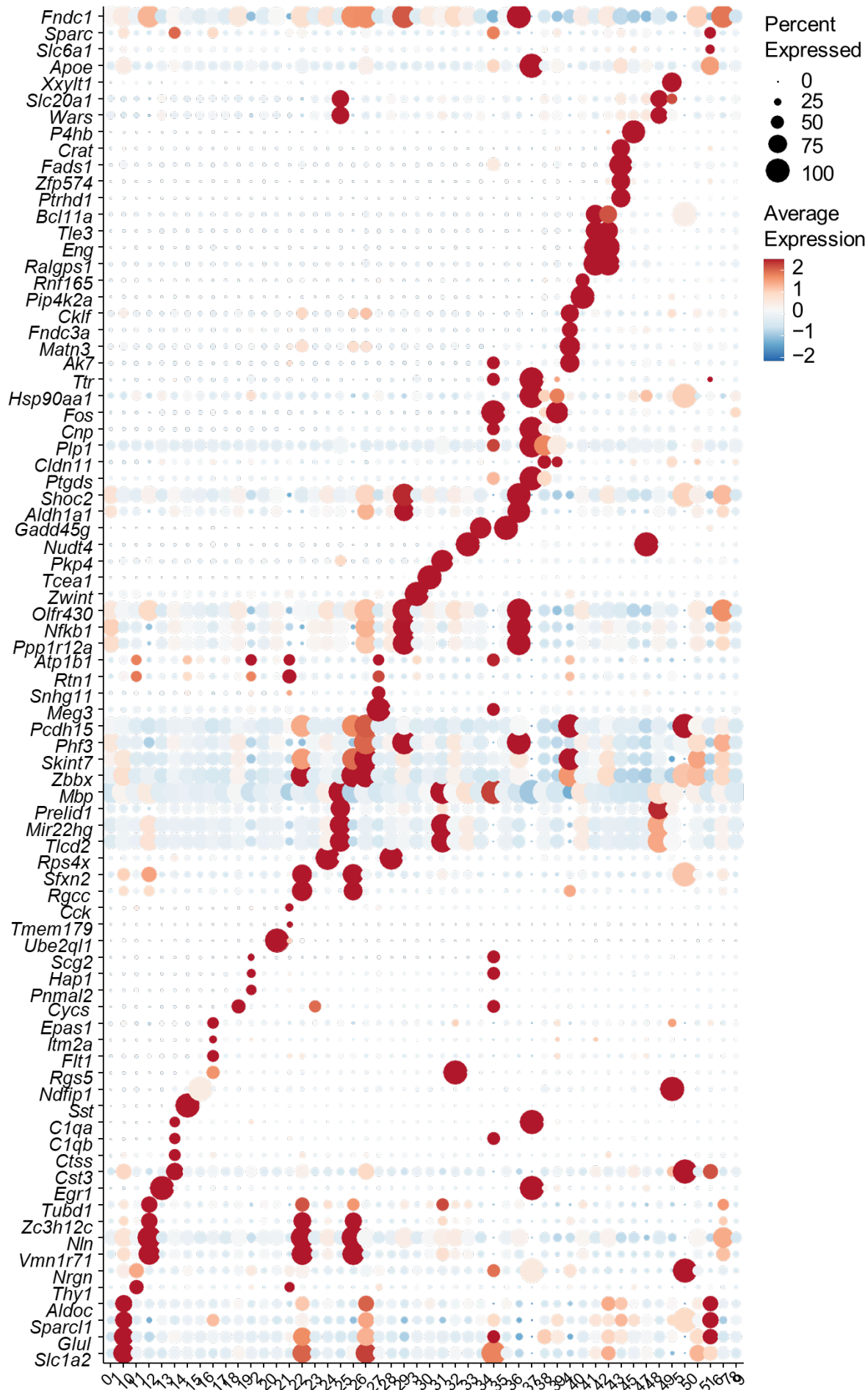

A

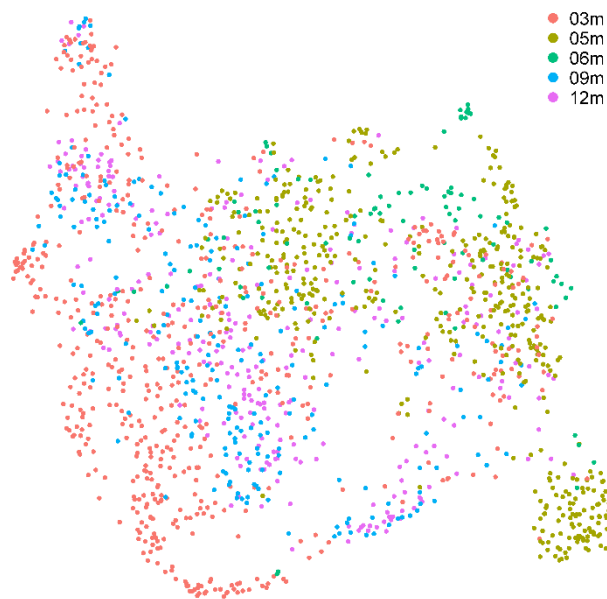

B

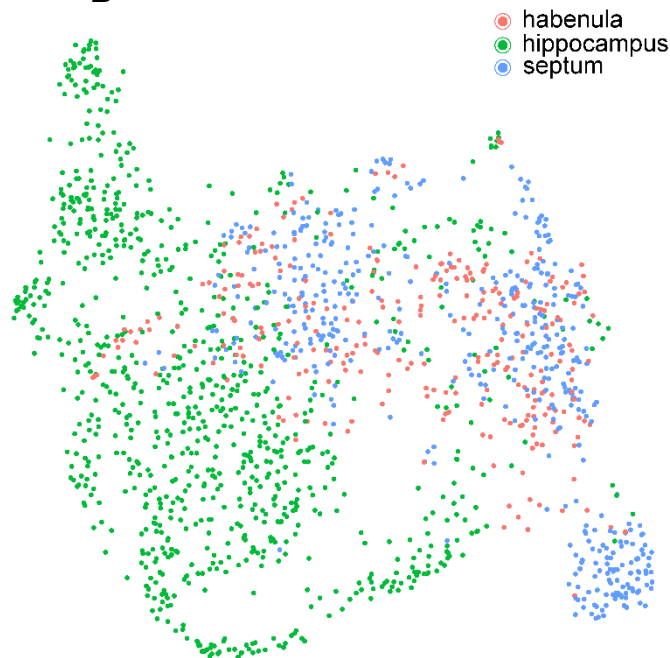

C

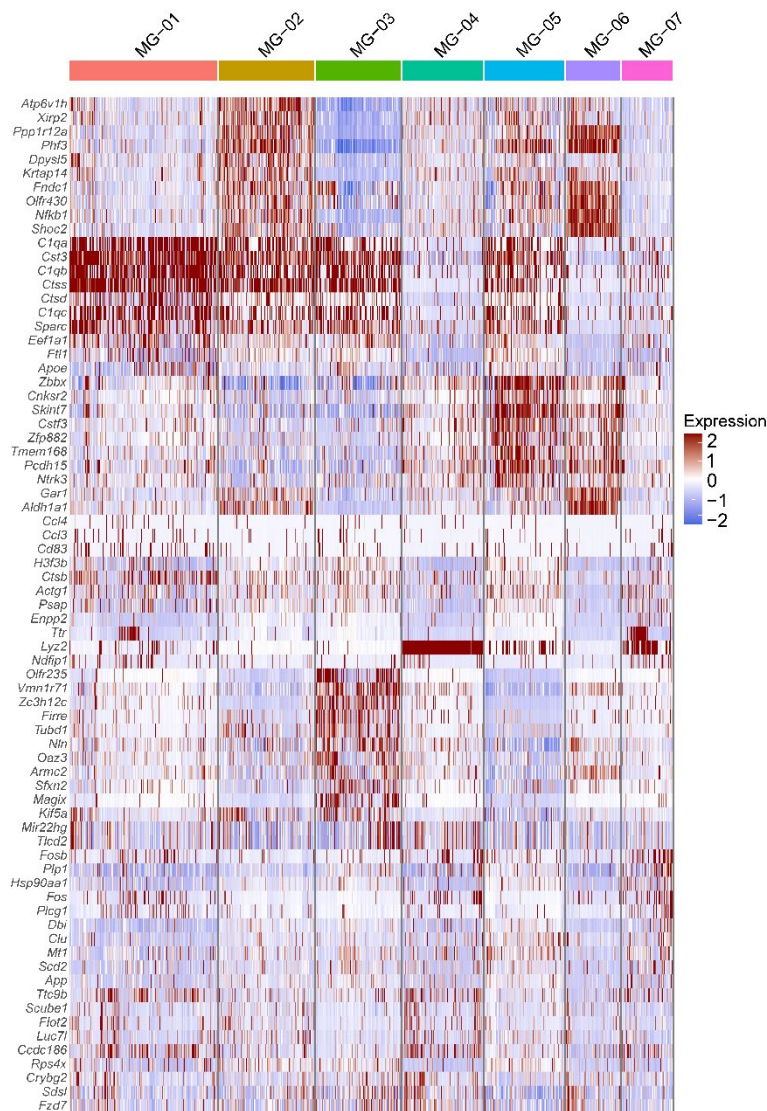

D

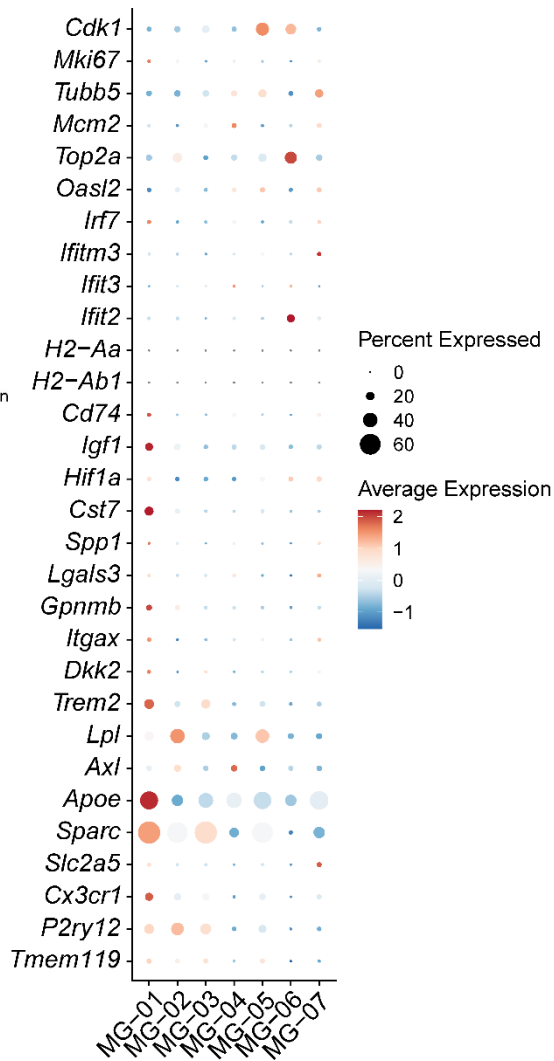

Supp. Fig. 3

A

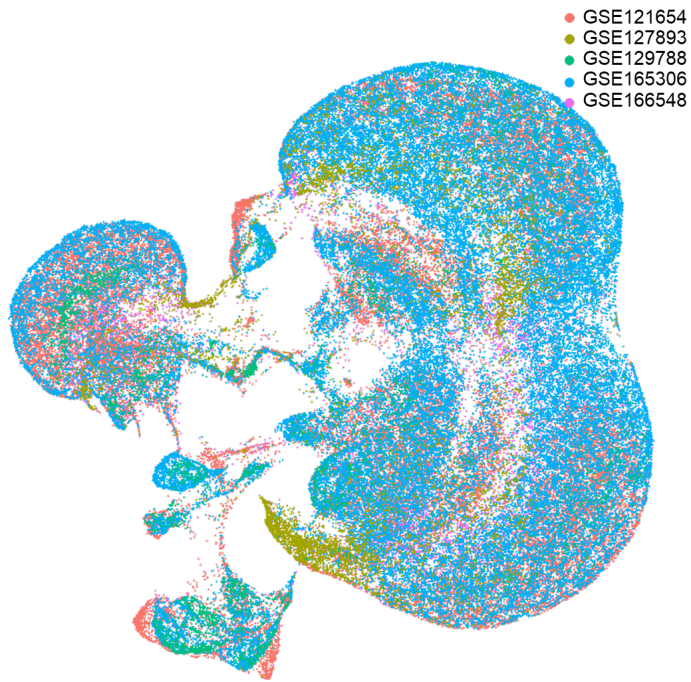

B

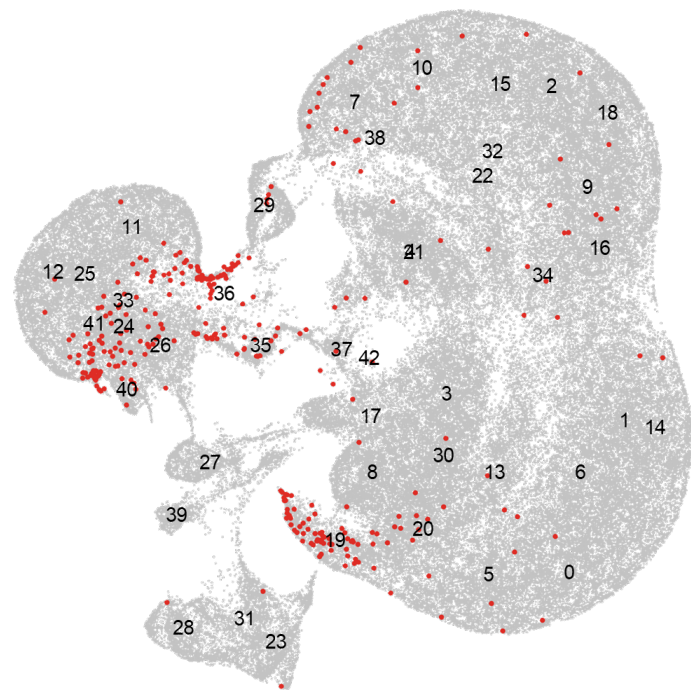

C

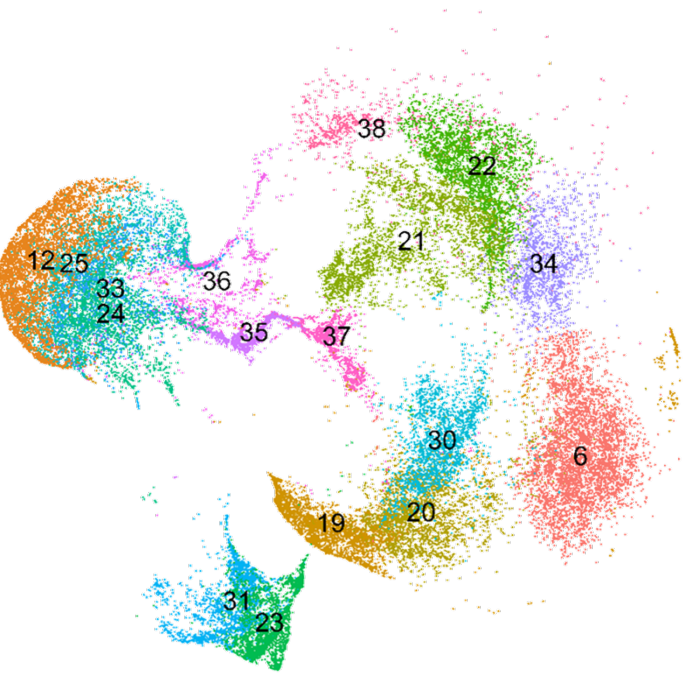

D

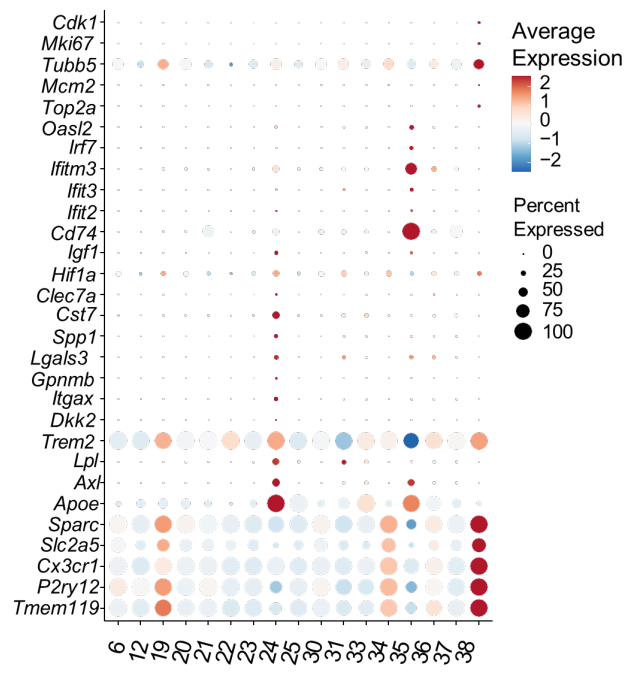

Supp. Fig. 4

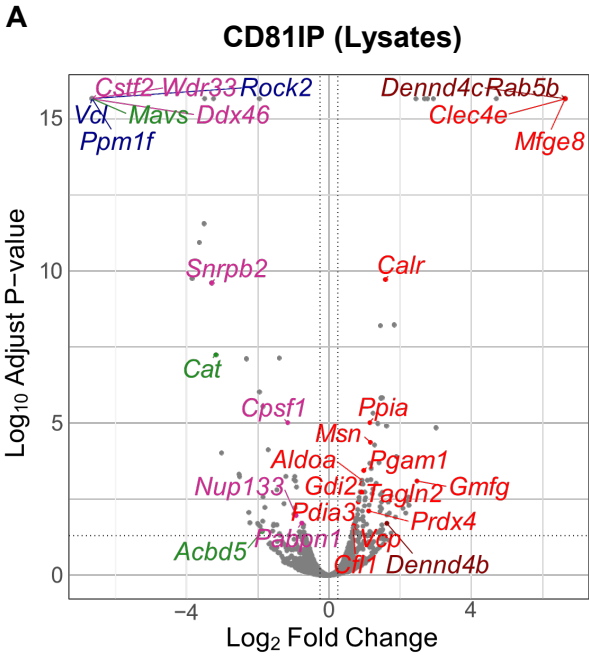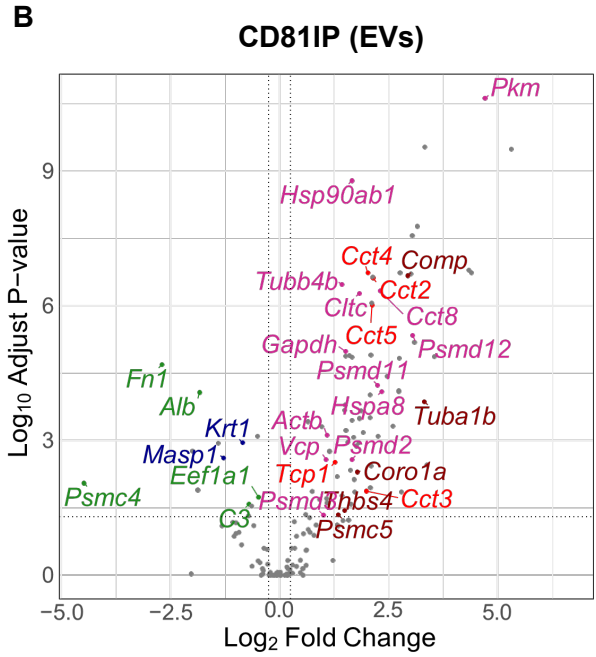

Supp. Fig. 5

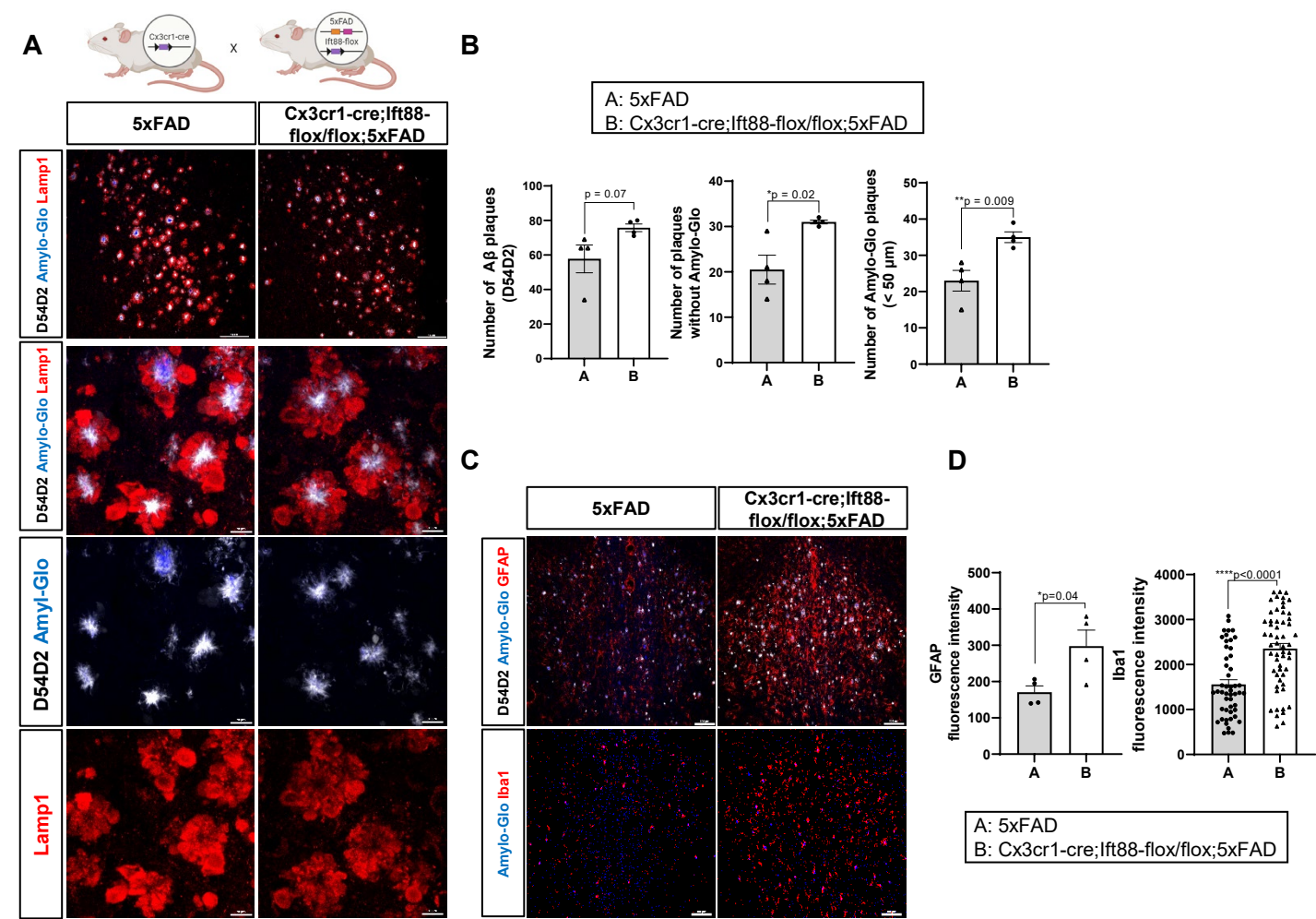

Supplement: Supplementary file 3 [file Presentation1.pdf]
